# Multidiscipline Applications of Triboelectric Nanogenerators for the Intelligent Era of Internet of Things

**DOI:** 10.1007/s40820-022-00981-8

**Published:** 2022-12-20

**Authors:** Xiaole Cao, Yao Xiong, Jia Sun, Xiaoyin Xie, Qijun Sun, Zhong Lin Wang

**Affiliations:** 1 Beijing Institute of Nanoenergy and Nanosystems, Chinese Academy of Sciences, Beijing, 101400 People’s Republic of China; 2 School of Nanoscience and Technology, University of Chinese Academy of Sciences, Beijing, 100049 People’s Republic of China; 3 School of Physics and Electronics, Central South University, Changsha, 410083 People’s Republic of China; 4 School of Chemistry and Chemical Engineering, Hubei Polytechnic University, Huangshi, 435003 People’s Republic of China; 5 Shandong Zhongke Naneng Energy Technology Co., Ltd., Dongying, 7061 People’s Republic of China; 6 School of Materials Science and Engineering, Georgia Institute of Technology, Atlanta, GA 30332 USA

**Keywords:** Triboelectric nanogenerator, Self-powered sensor, Internet of things, Artificial intelligence, Machine learning

## Abstract

In the era of 5G and the Internet of things (IoTs), various human–computer interaction systems based on the integration of triboelectric nanogenerators (TENGs) and IoTs technologies demonstrate the feasibility of sustainable and self-powered functional systems. The rapid development of intelligent applications of IoTs based on TENGs mainly relies on supplying the harvested mechanical energy from surroundings and implementing active sensing, which have greatly changed the way of human production and daily life. This review mainly introduced the TENG applications in multidiscipline scenarios of IoTs, including smart agriculture, smart industry, smart city, emergency monitoring, and machine learning-assisted artificial intelligence applications. The challenges and future research directions of TENG toward IoTs have also been proposed. The extensive developments and applications of TENG will push forward the IoTs into an energy autonomy fashion.

## Highlights

- Multidiscipline application of triboelectric nanogenerators (TENGs) for intelligent Internet of Things (IoTs) are summarized from the aspects of agriculture, industry, city, emergency monitoring, and artificial intelligence.
- Perspectives on the challenges and future research directions of TENGs in IoTs have been proposed.

## Graphical abstract

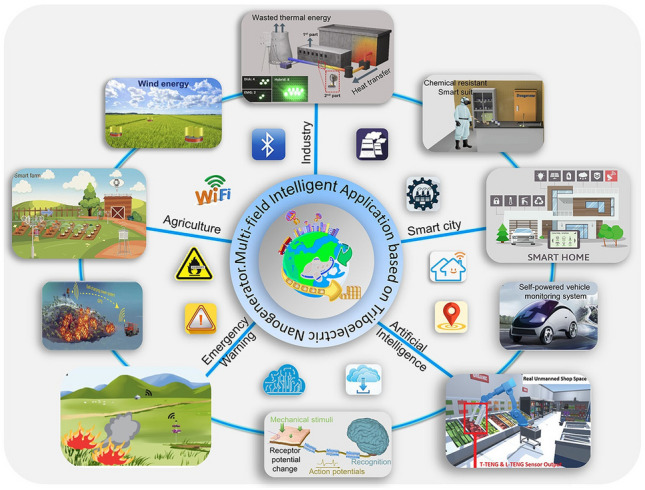

## Introduction

After years of academic research and commercial promotion on Internet of things (IoTs), various applications (e.g., smartphones, intelligent monitoring, home security systems, wearable electronic devices) have greatly improved human life in multidiscipline aspects. The concept of IoTs, defined as things (or people) connecting to the Internet through functional nodes, has rapidly expanded to various fields of intelligent transportation, smart environment, urban construction, industrial manufacturing, augmented reality (AR), virtual reality (VR), etc. [1]. Wireless sensor network is the core of IoTs, which is commonly composed of more than one billion sensors and electronic devices [2–5]. With the popularity and application of IoTs, the number of wireless sensor nodes rapidly increases, and the total power consumption of the electronic devices and batteries in IoTs network will be extremely high [6–8]. Regarding to the increasing demand for numerous sensors in the IoTs and the companied high-power-consuming issues, the self-powered sensors/systems without power supply have become the most promising and sustainable solutions.

TENG was first invented by Prof. Zhong Lin Wang in 2012 [6], which has been widely developed as high-entropy mechanical energy harvesters (ranging from wind energy [9–15], blue energy [16–20] to biomechanical energy [21–25]). TENGs have attracted wide attentions due to its low cost, diverse structures, stable output, high energy conversion efficiency, excellent environmental adaptability, eco-friendliness, etc. Various human–computer interactive systems based on the integration of TENG and IoTs technology have been demonstrated, which enables the sustainable applications of IoTs in environmental monitoring, smart farms, smart transportation, smart homes, smart industry, etc. The self-powered system embedded in IoTs effectively makes the living environment better and greatly changes the way of human production and life.

The fast development of artificial intelligence (AI) provides new possibility for data analysis through machine learning (ML) to reinforce the application of TENGs in IoTs [26, 27]. These subtle features hidden behind the real-time signal spectrum could be automatically extracted for AI applications, e.g., gesture recognition [28], texture recognition [29], object classification [30, 31], VR [32], and digital twins [33]. Besides, ML also provides an important data analysis tool for processing/analyzing the sensing signals detected by TENG, inspiring a promising research and development direction to the IoTs applications [34–37].

Recently, many excellent review articles have been published on the application of TENG in different and specific research fields. For instance, Liu et al. summarized the multi-domain applications of IoTs driven by advanced mechanical energy harvesters in the construction of smart city in recent years [38]. Zhou et al. discussed the application prospects of AI-assisted self-powered sensors in intelligent motion, security, touch control, and document management systems [27]. Sun et al. systematically introduced the current human–machine interactions (HMIs) from different application scenarios, such as wearable electronics, robot-related demonstrations, smart home, as well as the future development prospects of AI/haptic-feedback technology [39]. This review aims to comprehensively summarize the multidiscipline application of TENGs in five common scenarios of IoTs, including smart agriculture, smart industries, smart cities, emergency monitoring, and ML-assisted AI applications. Based on the literature reports, there are two main approaches to develop intelligent IoTs applications with TENGs. One approach introduces TENG sensors into currently existing components, such as the integration into tires to monitor tire pressure, into mechanical keypads for password recognition, into bearings for speed detection (or wear monitoring). The other route is to utilize/incorporate TENG into certain scenario as an original sensing system, e.g., temperature and humidity detection in smart farms and human–machine interfaces in smart homes. Finally, the challenges and future research directions to the construction of IoTs based on TENGs are discussed.

## Working Mechanism of Triboelectric Nanogenerator

**Fig. 1:**
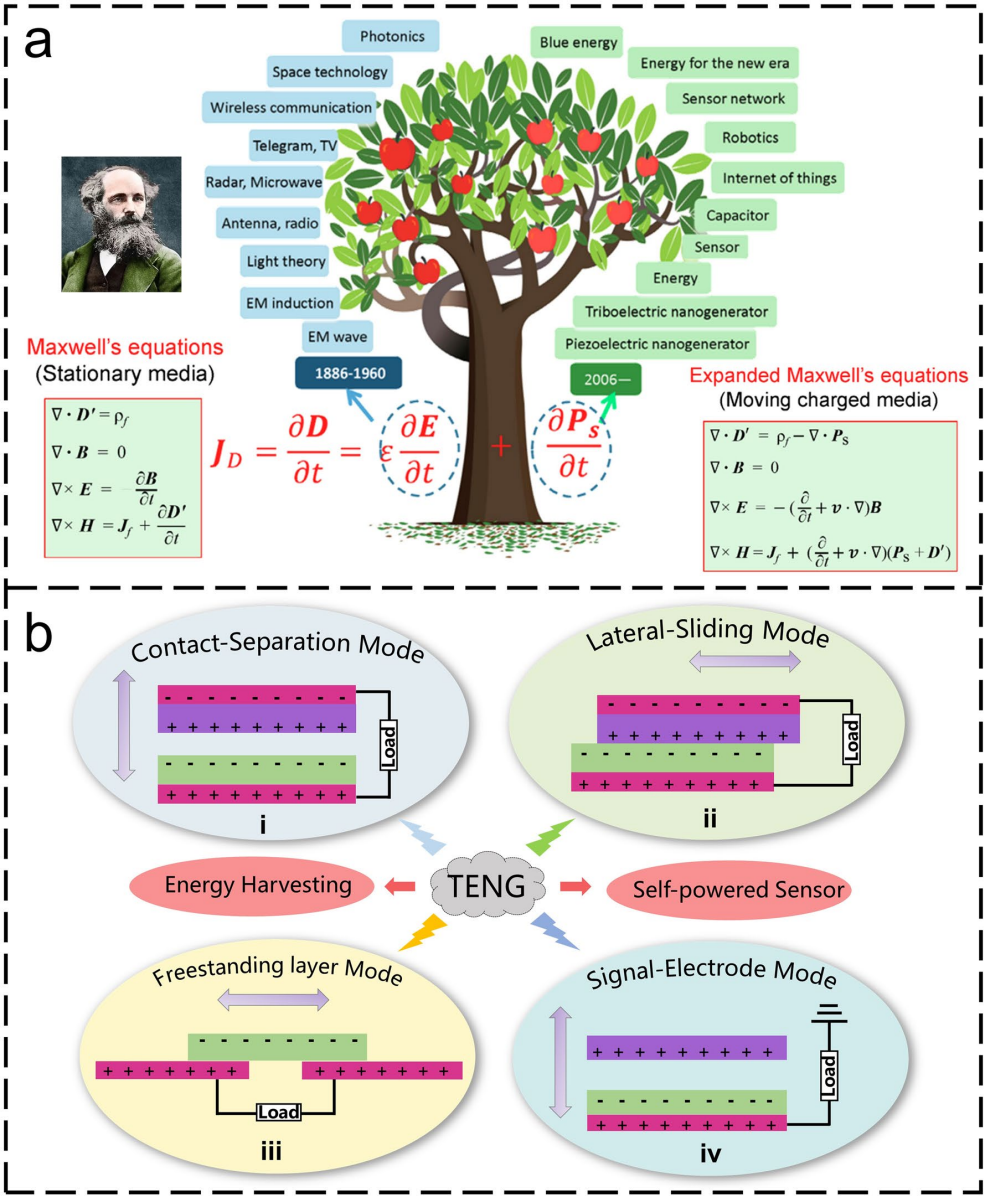
**a** A comparison of the Maxwell’s equations for stationary media and moving charged media [42]. **b** The basic operation modes and two main applications of triboelectric nanogenerators. **i** Contact-separation mode. **ii** Lateral-sliding mode. **iii** Freestanding mode. **iv** Single-electrode mode

Recently, the concept of displacement current has been applied for quantifying the power output of TENG (i.e., the energy widely distributed in our living environment with low quality, low amplitude, and even low frequency). The first TENG was invented by Wang’s research group in 2012, the main goal of which was to collect micro/nanoscale mechanical energy [40]. And the principle could be traced back to Maxwell’s displacement current. Wang et al. found that the second term $\frac{\partial P}{\partial t}$ in the Maxwell’s displacement current was directly related to the output electric current of the nanogenerator, indicating that TENGs were the applications of Maxwell’s displacement current in energy and self-powered sensors [41]. After that, Wang further developed and expanded Maxwell’s equations by including the polarization density term *P*_S_ in displacement vector (e.g., the electrostatic charges induced on medium surfaces by triboelectrification effect). On this basis, the fundamental theory for TENGs had been developed (Fig. 1a) [42, 43]. In addition to harvesting small-scale mechanical energy as a sustainable power supply for self-powered systems [44], TENG can also be used as self-powered sensors to convert mechanical energy into electrical output signals without external power supply.

According to practical application requirements for energy harvesting and self-powered sensing, four different working modes (or hybrid working modes) can be selected on demands, including vertical contact-separation (CS) mode, lateral sliding (LS) mode, freestanding triboelectric-layer (FS) mode, and single-electrode (SE) mode (Fig. 1b). In general, CS mode is applicable to the vertical contact-separation movement in a cycle such as the contact separation between the palm of the foot and the ground when walking [45, 46]. Similarly, LS mode is the friction of tangential motion. The CS and LS modes are usually used for energy harvesting, which are also beneficial for high-performance self-powered sensing [47, 48]. SE mode TENG with a simple structure is the most widely used for broad sensing scenarios. As for the FS mode TENG, it is mainly used for rotary motion [49, 50]. In real application scenarios, the appropriate working mode is often selected based on the comprehensive consideration of application scenarios, carrier types, and mechanical motion modes.

Take the contact-separation mode TENG as an example, the working mechanism of TENG is mainly explained according to the theory of triboelectric effect and electrostatic induction. When two dissimilar materials are in contact with each other, their surfaces will generate positive and negative charges due to contact electrification. When the two materials are separated by mechanical force, the positive and negative charges generated by contact electrification will also be separated. The charge separation will create a potential difference between the upper and lower electrodes of the material. The induced potential difference drives electrons to flow between the two electrodes through an external circuit. When the contact status changes periodically, opposite charges are generated on the two contact surfaces, resulting in continuous AC output.

**Fig. 2:**
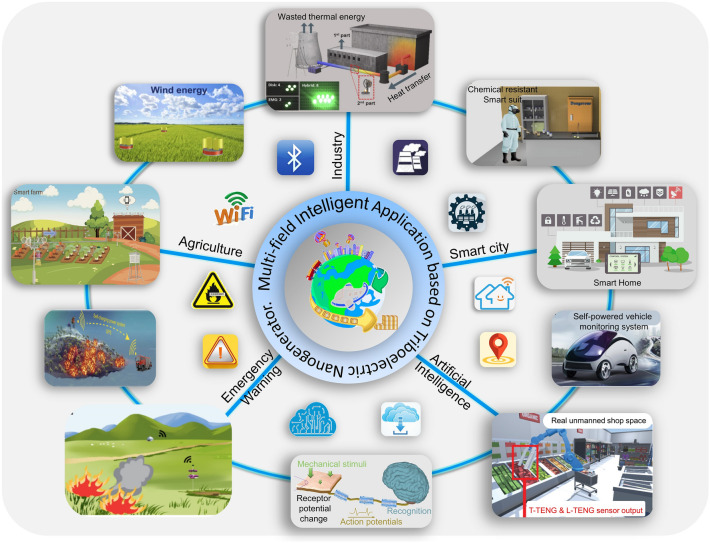
Multi-discipline applications of triboelectric nanogenerators for the intelligent era of Internet of things [29, 46, 68, 75, 80, 82, 129, 149, 150, 187]

At the same time, with the development of TENG, the applications of TENG are mainly divided into two categories, including energy harvesting and self-powered sensing system. For the self-powered sensing system based on TENG, it can be divided into two types. One is active sensor, which uses the signal generated by TENG itself as the sensing signal after external triggering [51–53]. This form of sensing system requires the TENG to have the highest output performance and energy conversion efficiency. For TENG devices, the surface charge density, which dominates output performance, is inevitably limited by the air-breakdown effect between two tribo-surfaces [54, 55]. TENG parallel-plate model has indicated that a thinner dielectric film, a larger dielectric constant, or a lower atmospheric pressure can lead to higher maximum surface charge density. Accordingly, relevant strategies (e.g., surface modification [56], air ion injection [57], external charge pumping [58, 59]) have been developed to increase the charge density. And ultra-high energy conversion efficiency of 48% could be achieved by utilizing a novel voltage balance bar design in liquid lubrication and charge space-accumulation effect [60]. These studies are significant to promote the commercialization of TENG-based self-powered systems. The other is that TENG acts as an energy harvester to provide electrical energy for the sensor [61]. Here, we will summarize the application of TENGs in the intelligent IoTs from five aspects: agriculture, industry, city, emergency monitoring, and ML-assisted AI applications (Fig. 2).

## TENG Applications in Smart Agriculture

**Fig. 3:**
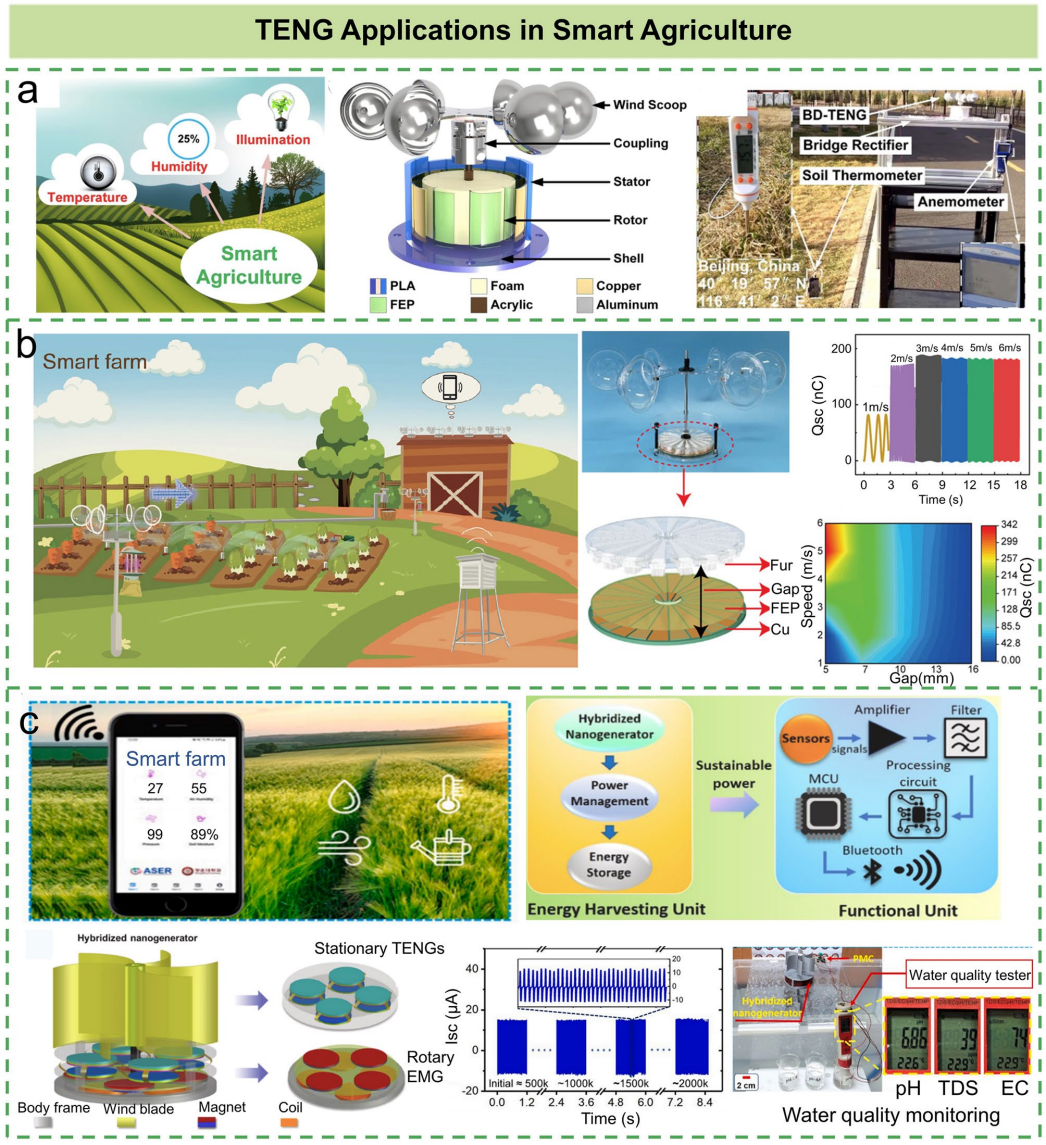
Application of triboelectric nanogenerators in smart agriculture. **a** Three essential elements of smart agriculture, the illustration of the BD-TENG structure, and application scenario of collecting natural breeze energy to power soil thermometer [62]. Copyright 2021 Elsevier Ltd. **b** Schematic diagram of the proposed SCR-TENG in the smart farm, the detailed structure, and electrical performance of the TENG [68]. Copyright 2021 Wiley–VCH GmbH. **c** System architecture of the self-powered wireless smart-farm monitoring (SWSFM) system powered by the CMTUr-HNG and the schematic diagram of ultra-robust hybrid nanogenerator (CMTUr-HNG) triggered based on non-contact mode [75]. Copyright 2021 Elsevier Ltd

Agriculture is the primary element to support the construction and development of the national economy. Traditional agriculture is often affected by natural disasters so that the agricultural production is less efficient. Therefore, smart agriculture combining traditional agriculture with modern technology is urgent to effectively deal with these difficulties [62]. Besides, appropriate temperature, humidity, and light intensity are important parameters to be monitored to ensure the favorable growth of crops and plants. In recent years, TENG-based energy harvesters have been widely used to harvest surrounding mechanical energy in farming environments and directly power the agricultural sensors (Table 1). Among different kinds of mechanical energy, natural wind energy has the advantages of abundance, wide distribution, renewable, no pollution, etc., which is a good choice to energy-harvesting and powering agricultural sensors [63, 64]. For instance, Li et al. designed a breeze-driven triboelectric nanogenerator (BD-TENG) to provide energy for smart agricultural production systems [62]. The detailed structure of the BD-TENG is shown in Fig. 3a, which is composed of wind scoops, coupling, rotor, stator, and shells. The natural wind is collected by the wind scoop and drives the FEP film to produce sliding friction against the copper electrode, thereby converting the natural wind into electricity. The output performance of the BD-TENG is 330 V, 7 μA, 137 nC, and the peak power is 2.81 mW at the wind speed of 4 m/s. The experiment results show that the collected natural wind energy can supply power to soil thermometers and multiple series-connected light-emitting diodes (LEDs), which verifies its potential application in smart agriculture. The direct contact between the two friction materials helps to generate more triboelectric charges and higher output power density, but it may lead to durability problems [65]. Previous work has demonstrated that TENG with soft-contact mode is an effective strategy to improving durability [66, 67]. Accordingly, Han et al. fabricated a soft-contact rotary TENG (SCR-TENG) for harvesting low-speed wind energy using elastic and soft rabbit hair as a typical triboelectric material [68]. The schematic illustration of SCR-TENG in smart farm is shown in Fig. 3b. On the one hand, using flexible fur as the triboelectric material to fabricate TENG can not only help to inject more positive triboelectric charges into the dielectric surface, but also greatly reduce material wear. On the other hand, the external driving force can be greatly decreased, while the output power density (11.9 mW) and the durability of the equipment can be improved at the same time (no obvious decay in the transferred charges for 480,000 operation cycles). They successfully demonstrated the TENG applications in night direction indication, insect capture, soil moisture detection, ambient temperature/humidity detection, and signal transmission.

**Table 1:** Application of TENG in smart farm

Refs.	Energy sources	Current (μA)	Power density (mW)	Sensor types
Li et al. [62]	Wind	7	2.81	Soil thermometer
Han et al. [68]	Wind	15	11.9	Temperature, humidity
Rahman et al. [75]	Wind, blue energy	26	5.39	Hygro-thermograph
Li et al. [79]	Wind and rain	73	6.35	Temperature, humidity and soil pH

Although TENGs have been proven to be an effective strategy to harvesting irregular and low-frequency mechanical energy (e.g., wind, water) in smart farm applications [69], the low output power of TENG often hinders its application in autonomous IoT sensors and self-powered wireless sensor networks [59, 70]. Hybrid nanogenerators with different types of energy-harvesting strategies provide an effective means to solve this problem. The electromagnetic generator (EMG) has a higher output current, while TENG exhibits higher output voltage under the same mechanical input. Therefore, the EMGs and TENGs can be hybridized into a common system to complement each other and improve the overall output performance [71–74]. Rahman et al. reported an ultra-robust rotary hybrid nanogenerator (Ur-HNG) based on EMG with soft magnets coupled with non-contact mode TENG [75]. As shown in Fig. 3c, the Ur-HNG includes an array of four CS mode TENGs and rotating EMGs, which play complementary roles in the output performances and exhibit a maximum power density of 1568 mW Kg^−1^ (the load power per unit mass) and an average power density of 386 mW Kg^−1^ with the matching load resistance. Based on the harvested wind energy, a real-time self-powered wireless smart-farm monitoring (SWSFM) system is developed by integrating energy-harvesting unit and functional unit. The SWSFM system is composed of hybrid sensor BME280, operational amplifier, filtering circuit, signal processing circuit, microcontroller unit (MCU), and Bluetooth wireless communication unit. The sensors are used to collect information about vital parameters of smart farm, such as humidity, temperature, and soil moisture content. The transmitted sensor data can be received via Bluetooth-enabled devices (e.g., smartphones) to monitor the environmental conditions of smart farms. Relevant studies have shown that the external high electrostatic field can not only be used to accelerate seed germination and plant growth [76, 77], but also improve the ion migration in the soil for better nutrient absorption [78]. Li et al. reported a wind and rain energy-driven electric stimulation system based on all-weather TENG for improving crop yields [79]. On the one hand, through self-powered treatment by TENG-derived high-voltage electric field, the system can increase the germination speed of pea seeds by ~ 26.3% and the yield of peas by ~ 17.9%, which has demonstrated that it can convert surrounding mechanical energy into electricity to improve the crop yields. On the other hand, by harvesting the energy from environmental wind and raindrops, the all-weather TENG can be used to drive various agricultural sensors for optimizing the growth conditions of plants.

## TENG Applications in Smart Industry

The industrial IoTs focus on the four application scenarios: monitoring and optimization of the on-site industrial production process, optimal resource allocation, collaboration to strengthen production safety, and costs reduction. Numerous sensors play various roles in modern factories, including provision of data for process control, quality assessment, production monitoring, worker safety, etc. In recent years, the application of TENGs in industrial scenarios has attracted extensive attentions. This section mainly describes the application of TENG-based energy harvesters and self-powered sensation in industrial scenarios.

**Fig. 4:**
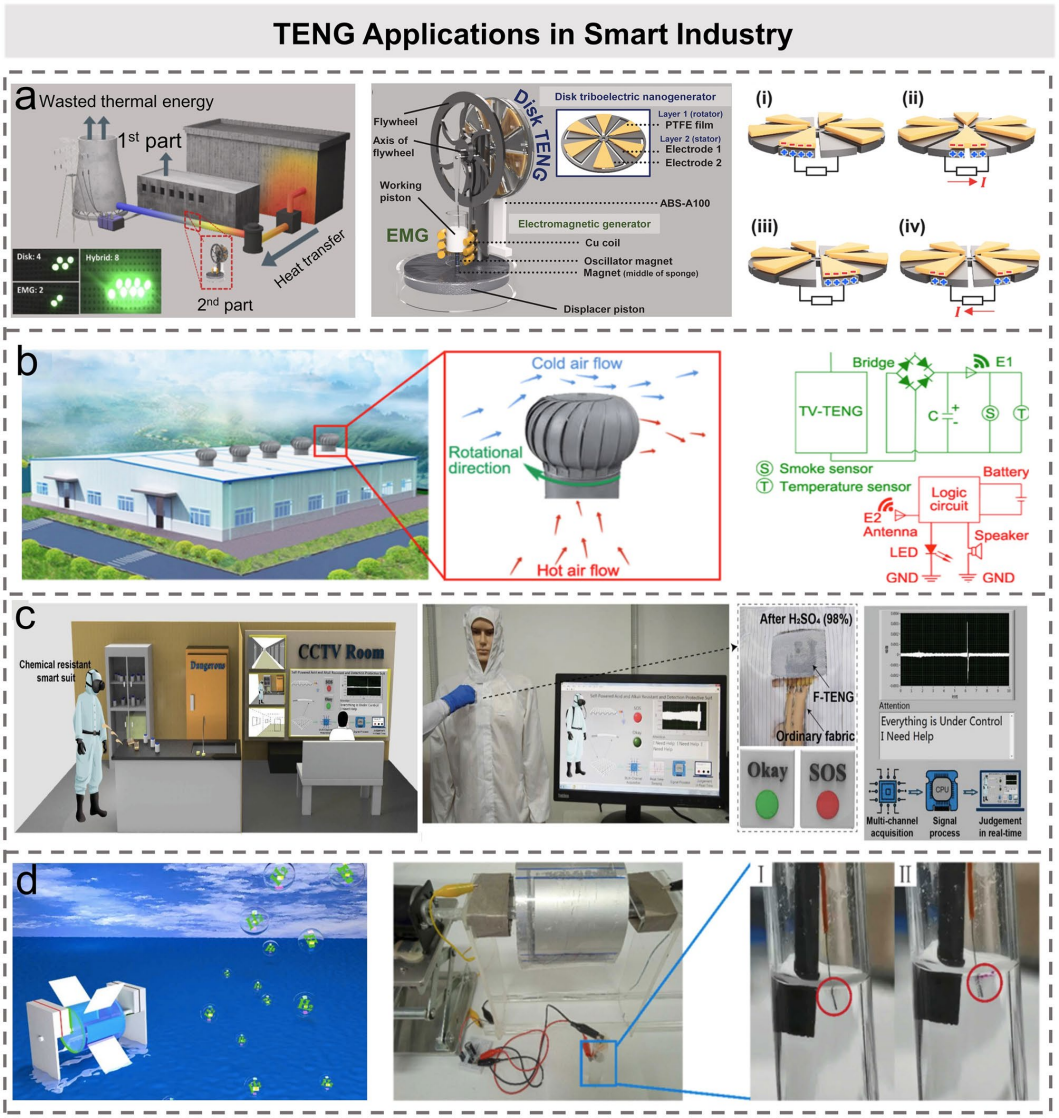
TENG applications in smart industry. **a** Schematic of the proposed heat recovery system, structure of the LDT Stirling engine, the disk TENG, and the EMG [80]. Copyright 2021 Elsevier Ltd. **b** Schematic diagram of TENG tilt sensor applied to ship attitude sensing and experimental apparatus, and circuit diagrams and design of the self-powered system [81]. Copyright 2021 American Chemical Society. **c** Application of the smart chemical protective suit in biological movement energy harvesting and self-powered safety monitoring system [82]. Copyright 2021 Wiley–VCH GmbH. **d** Schematic diagram of seawater self-powered electrolysis and TENG test system [87]. Copyright 2021 Published by Elsevier Ltd

With the rapid development of modern industry, human demand for energy is also increasing. However, a large amount of high-entropy energy cannot be reused and wasted during the production process, especially the enormous heat dissipation. In order to solve this problem, Yun et al. proposed a hybridized energy harvesting system (HEHS) to harvest the wasted thermal energy for industrial applications [80]. The HEHS is composed of a disk TENG and an EMG as a whole to be installed in the commercial low-temperature differential (LTD) Stirling engine. The structure of the LTD Stirling engine, disk TENG, and EMG is shown in Fig. 4a. According to the Stirling cycle, the difference in temperature causes the movement of the piston to realize the conversion of thermal energy to mechanical energy, and EMG is set simultaneously during the up and down movement of the piston. At the same time, the movement of the piston drives the movement of the flywheel, and the disk TENG can generate AC output due to the contact electrification between PTFE and aluminum electrodes. During this process, the hybridized disk TENG and EMG realize the conversion from mechanical energy to electrical energy assisted the wasted heat recovery. When the HEHS was operated, the advantages of EMG and TENG complement each other to obtain high output voltage and high output current. When the same heat energy was applied to each nanogenerator, the EMG, TENG, and HEHS can generate the electrical energy of 1.63, 8.55, and 23.65 μJ, respectively. The results demonstrated that the HEHS has potential application in heat recovery systems in industrial field. In addition to TENG for heat recovery, it is reported that TENG can also be used for self-powered modern industrial monitoring. Zhang et al. designed a turbine vent TENG (TV-TENG) on the roof of buildings to harvest irregular wind energy for self-powered environmental sensing system [81]. On the one hand, TV-TENG collects irregular wind energy in the environment for power supply of low-power sensors and other related microelectronic devices in industrial production. On the other hand, TV-TENG, a set of wireless transmitters and receivers, and a 330 μF capacitor constitute a self-powered wireless early warning system for monitoring production safety. The circuit diagram and design of the self-powered system are shown in Fig. 4b. Depending on the outdoor windy environment, the capacitor can be quickly charged to reach the operating voltage of the monitoring system in a short time and subsequently implement the real-time monitoring process. In addition to monitoring the status of industrial facility and production, the prevention of laboratory accidents and special workshop accidents is also very important during the production process. Ma et al. designed a smart protective laboratory-coat based on a fabric TENG composed of PTFE yarns, which has four main functions for self-powered monitoring during the extreme working environments [82]. First, it is woven from PTFE yarn, which has good acid and alkali resistance compared with ordinary fabrics. Second, it can be used for self-powered chemical-leakage monitoring, which can produce distinct signals when chemical droplets contact the hydrophobic yarn in core–shell structure. Third, the smart laboratory-coat can provide vital signs and motion monitoring to detect operator’s status in hazardous work environment. Fourth, as shown in Fig. 4c, tapping single-electrode TENG yarn on the protective clothing can realize real-time remote alarm.

TENG has also been reported for production line management. Gu et al. proposed a particle transport-based TENG (PT-TENG) for self-powered mass flow detection and explosion warning. It is well known that pneumatic conveying of particles has been widely used in food industry [83], petrochemical industry, and other processing and electronic industries [84, 85]. PT-TENG can be used as a self-powered detector to monitor the mass flow of particles in a timely manner. Moreover, a self-powered explosion-proof alarm device can be further developed on this basis to effectively guarantee the safety of the factory. Chen et al. proposed a pump-switch TENG (PSW-TENG) for real-time wireless sensing of plant production to monitor the metal products on the conveyor [86]. The structure of PSW-TENG is composed of pump TENG and main TENG, which are two typical freestanding rotating TENGs. (Pump TENG is used to improve the performance.) To obtain self-powered wireless sensing information, by integrating the inductance coil with PSW-TENG, the pulse signal from PSW-TENG is modulated into a resonant signal by LC oscillating circuit for wireless transmission. Due to the influence of metal on the resonant frequency of oscillation circuit, they proposed a self-powered metal composition detection/monitoring system combining PSW-TENG with the electromagnetic sensor, which can be used in factory pipeline for checking the types of tin and calculating the quantity of products. It can detect the height and horizontal position of the metal parts (which pass under the sensor) to identify the products and count the number of products, which is very suitable for the management of the production line.

The excellent electrical output of TENG can also be used for seawater electrolysis. Zhu et al. designed a pulsed TENG that could continuously harvest energy from water and wind for self-powered seawater electrolysis [87]. The structure and performance of TENG are illustrated in Fig. 4d, which shows a high current output of 24 μA after rectification. The production of hydrogen by TENG electrolysis of saturated salt was successfully demonstrated, showing the potential application in seawater electrolysis.

**Fig. 5:**
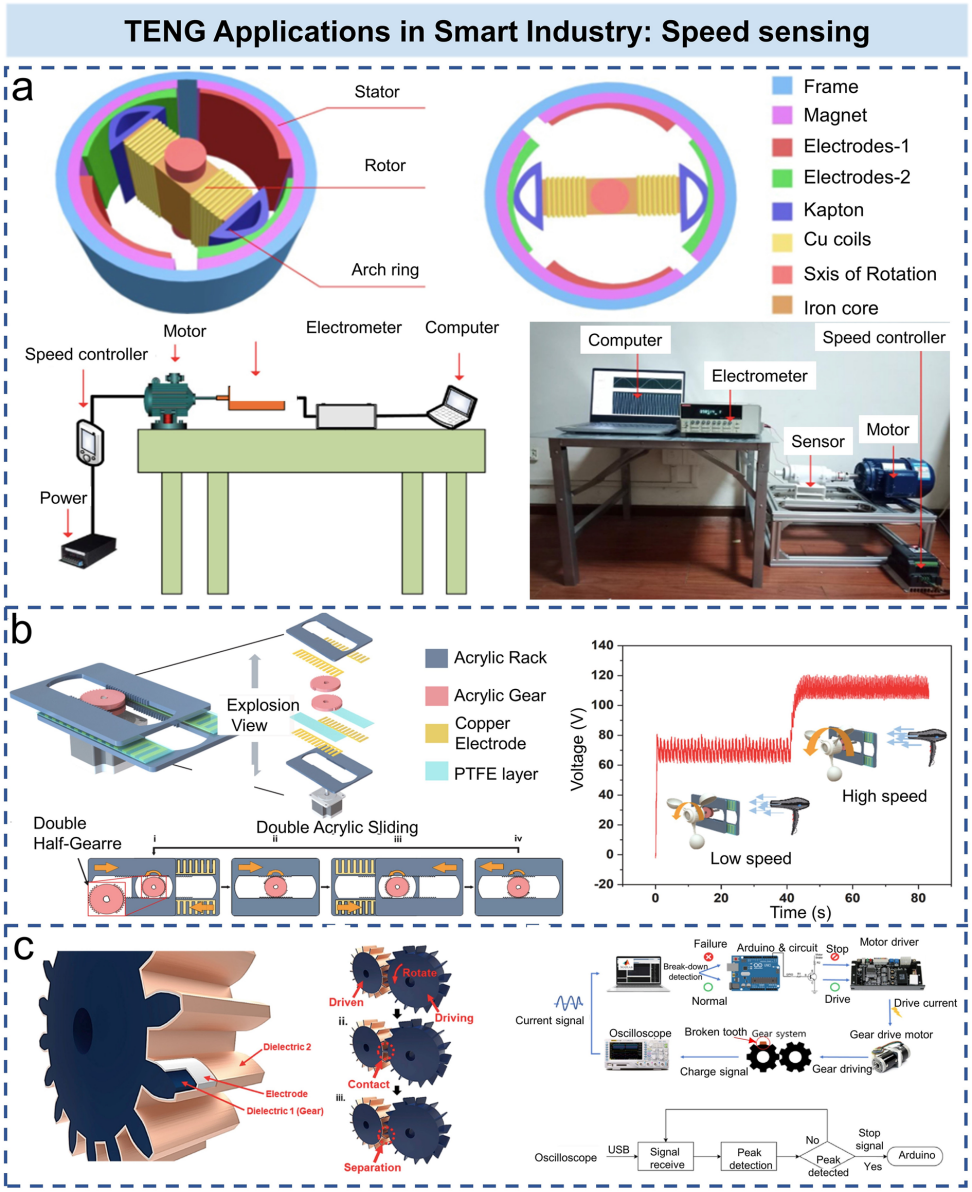
TENG applications in smart industry: speed sensing. **a** Schematic diagram of drill pipe sensor structure and testing devices [89]. Copyright 2021 Elsevier B.V. **b** 3D structure diagram of the self-powered sensor and actual use of impeller input for detecting low-speed and high-speed wind [92]. Copyright 2021 Elsevier Ltd. **c** Modification of the gear surface by continuous coating of conductive and dielectric materials, configuration of gear box operation condition monitoring system [93]. Copyright 2020 Elsevier Ltd

In modern industrial equipment, sensors and detectors are an indispensable part. In recent years, speed sensors based on TENG have been widely used in various industrial scenarios, such as drill pipes, speed sensors, gear condition monitoring, and smart bearings [88]. Figure 5a shows the drill pipe rotation speed sensor based on triboelectric–electromagnetic hybrid nanogenerator [89]. The TENG and EMG can be used as sensor and energy harvester independently with high reliability and sensitivity (measurement range at 0–1000 rpm with the sensitivity of 0.033 Hz rpm^−1^). Compared with conventional rotary TENG [90, 91], Lin et al. utilized the hybrid NG to implement the synchronous sensing of speed, displacement, and acceleration through the relative sliding of two rack-shaped acrylic plates (as shown in Fig. 5b) [92]. They have successfully transformed rotary motion into linear reciprocating sliding by using the gears in conjunction with the rack-shaped acrylic plates. The voltage sensitivity of the angular velocity for this self-powered sensor is 580 mV rpm^−1^, which has strong immunity to environmental noise. Gears are also commonly used in various industrial fields as effective mechanical power transmission components. When gears are used for power transmission, the contact and separation between the tooth pairs of meshing gears continue to occur, resulting in triboelectric signals. Accordingly, Ra et al. modified the gear in combination with TENG, and the generated triboelectric signal could reflect the in situ state of gears meshing in real time [93]. Based on this principle, they designed a gearbox operation state monitoring system (Fig. 5c). When the system is running, the generated triboelectric signals are measured in real time and transmitted to a laptop computer, which is subsequently analyzed by a monitoring algorithm to determine the state of the gearing.

**Fig. 6:**
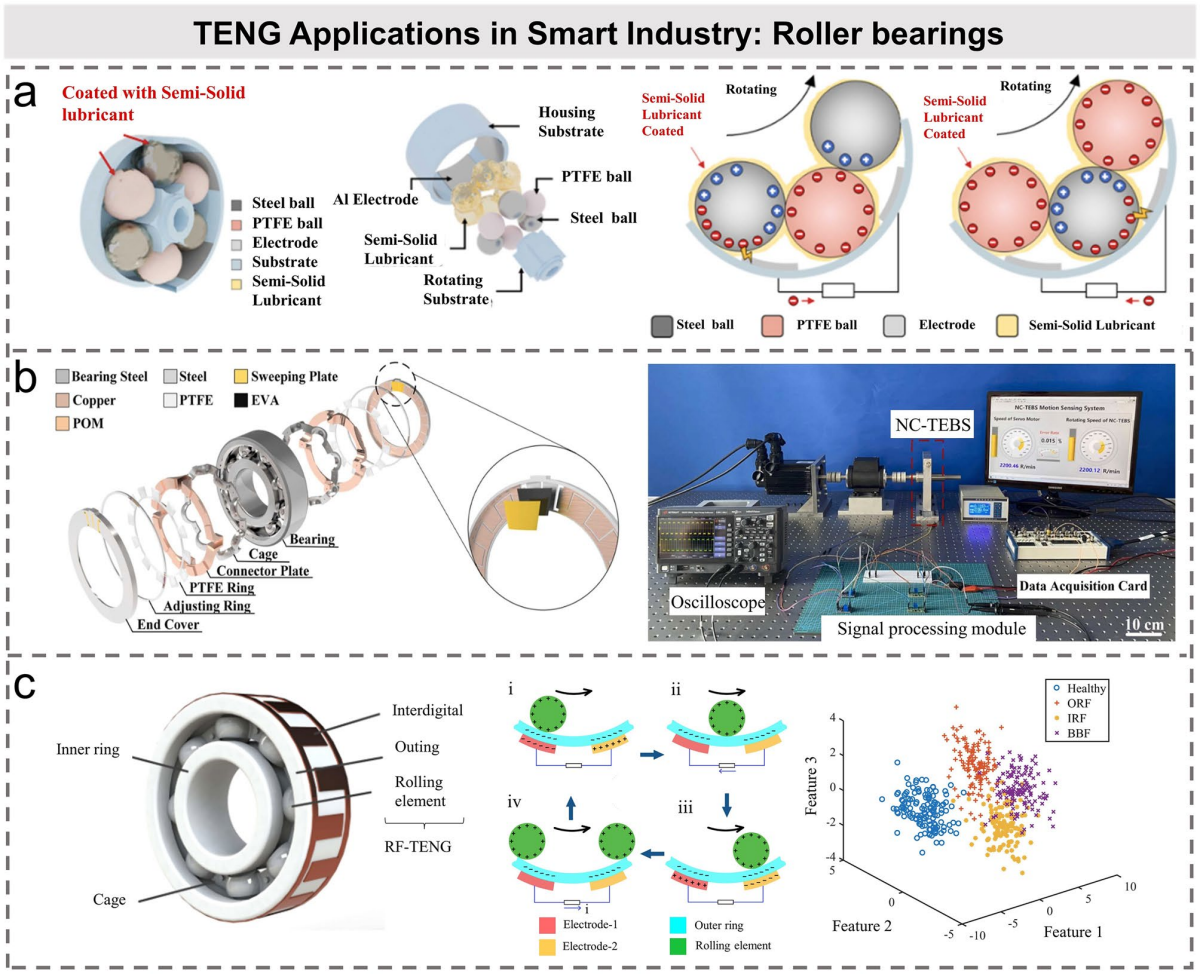
TENG applications in smart industry: roller bearings. **a** Schematic diagram of the principle and working mechanism of TENG-type ball bearing with semisolid lubricant [100]. Copyright 2021 Elsevier Ltd. **b** Structural decomposition diagram of NC-TEBS and real-time sensor monitoring and testing system using NC-TEBS [105]. Copyright 2021 Elsevier Ltd. **c** Structure diagram and working mechanism of rolling-independent mode TENG (RF-TENG); linear discriminant analysis (LDA) is used to extract features and reduce dimensions of the raw data [106]. Copyright 2021 Elsevier Ltd

Various sensors are mainly used to monitor the state of industrial equipment to maintain the normal operation. As an important mechanical part, rolling bearings are widely used in modern industrial equipment. On the one hand, the friction of the contact surface inevitably occurs during the operation of bearings. The rolling bearings can produce mechanical energy during the working state, including vibration and rotation energy [94]. Accordingly, as shown in Table 2, TENG based on bearing structure has been proposed and successfully utilized as speed sensor [95–99]. Song et al. designed a ball-bearing TENG, and the use of commercial semisolid lubricants improved both the mechanical life and electrical output of the system (Fig. 6a) [100]. On the other hand, the operation state of bearing directly affects the working performance, reliability, and service life of the electrical equipment [101, 102]. With the rapid development of modern industrial technology, higher requirements are put forward for the speed and service life of rolling bearings. However, the skidding phenomenon easily occurs at high speed, which is one of the most common failure forms of rolling bearings. It may cause scratches, wear, reduce the rotation accuracy of bearing, and significantly affect the working performance and service life of bearings [103, 104]. In order to avoid serious equipment damage and consequent major economic losses, it is very essential to monitor the skidding rate of rolling bearing. Therefore, Xie et al. developed a non-contact triboelectric bearing sensor (NC-TEBS) to monitor the speed and skidding rate of bearing (Fig. 6b) [105]. The sensor is integrated with the bearing and has an ultra-wide working speed range of 10–5,000 rpm, which can monitor the bearing speed at low-speed conditions and the bearing skidding rate at high speed with light load. In order to better diagnose the fault of rolling bearings, Han et al. glued flexible interdigital electrodes to the outer ring of rolling bearings to form a rolling TENG [106], as shown in Fig. 6c. The variation of output current amplitude and frequency with rotation speed is analyzed. Based on the model of output signals, a variety of classification algorithms are used for ML, and the fault characteristic frequency of the TENG output current is obtained when the outer ring, inner ring, and ball part failed. The achieved accuracy is more than 92%, which proves that TENG has a good application prospect in rolling-bearing fault diagnosis.

**Table 2:** TENG-based bearing structure for speed sensing

Refs.	Accuracy	Error rate	Range (rpm)	Application
Li et al. [96]	2.5°	0.1%	100–1000	Inspecting bearing flaws
Choi et al. [97]	15°		20–600	Bearing monitor
Xie et al. [98]	6°	0.1–0.3%	10–1000	Rotational speed
Xie et al. [105]	14°	0.25%	10–5000	Bearing monitor
Zhang et al. [201]	1.5°	0.2–0.8%	10–1000	Speed, angle monitor

## TENG Applications in Smart City

As an important scenario of IoTs, smart cities have greatly improved the travel fashion and living quality of human beings. The concept of smart city is becoming a reality thanks to the fast development of advanced sensing networks in IoTs with reliability, high efficiency, and low-power consumption [107, 108]. TENGs can be readily installed in distribution style to scavenge multi-category mechanical energy and implement various sensing applications in smart cities. In this section, the TENG applications in smart city is mainly sorted into smart homes and smart transportation, including driving behavior monitoring, traffic and railway monitoring, structural health monitoring of bridges and buildings, etc.

### Smart Home

**Fig. 7:**
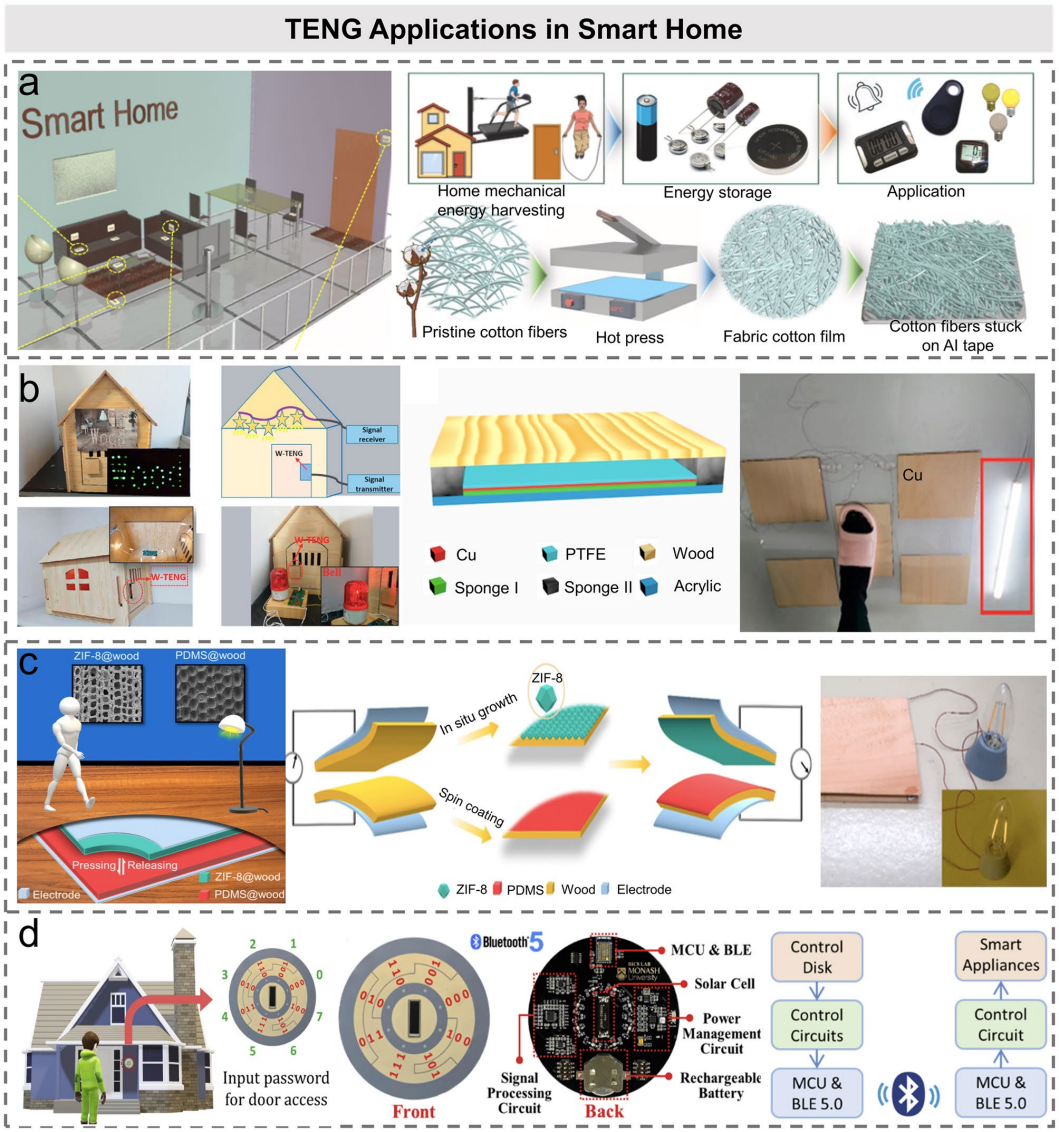
TENG applications in smart home. **a** Schematic illustration of household mechanical energy, energy storage, and their applications [46]. Copyright 2020 Elsevier Ltd. **b** Application of W-TENG in smart home, and the schematic diagrams of W-TENG [110]. Copyright 2020 Elsevier Ltd. **c** Practical smart home applications of all wood-based TENG [147]. Copyright 2021 Elsevier Inc. **d** Illustration of the password authentication access control system. A password is required to enter the door and access control system [47]. Copyright 2020 Elsevier Ltd

As an important constituent part in the construction of smart city, the smart home has attracted extensive attentions in recent years. As shown in Fig. 7a, Graham et al. fabricated a TENG suitable for smart home from plastic and electronic waste that are common in households [46]. The TENG can sense the motion state of the human body and collect energy generated by daily activities. Furthermore, the authors also developed a peak voltage detection circuit to be combined with the TENG and used for motion sensing. Applying energy harvesting and active sensor technology based on TENG to traditional floors and tiles can record the information of human walking [109–112]. Hao et al. manufactured a naturally biodegradable wood-based TENG, with a maximum output power density of 158.2 mW m^−2^ at a load resistance of 50 MΩ (Fig. 7b) [110]. The TENG was also demonstrated for self-powered light switches and self-powered doorbells to save unnecessary power consumption. Moreover, it can be designed and installed on the stage wooden floor to track and record the movements of dancers, through which the dancers can control the lights through the floor under their feet. Sun et al. prepared two wood materials with superior friction properties by chemical modification to fabricate an all-wood TENG (Fig. 7c) [113]. The voltage output of TENG prepared from modified wood is 80 times higher than that of pure wood. In addition to supplying power for simple equipment, the proposed TENG can also supply power for smart electrochromic windows to adjust sunlight transmittance and save energy for the indoor lighting.

Smart windows in house are another importance research area to intelligent life. Wang et al. reported a triboelectric potential modulated smart window, which combines rotary FS mode TENG and polymer network liquid crystal (PNLC) units [114]. When applying an alternating electric field generated by TENG, the transparent smart window will become opaque immediately. The switching state between ultra-transparent and ultra-blur has been used for privacy protection. Recently, their group has further developed a smart window based on elaborately tailored cholesteric liquid crystal and TENG [115]. The smart window can be momentarily powered up to remain hazy and then disconnected and restored to a transparent state by pressure loading. Similarly, Yeh et al. proposed a self-powered smart window system with an electrochromic reaction driven by TENG operating under wind or raindrops [116]. In addition, Tan et al. presented a rotary FS mode TENG for driving the anti-fog device [117]. Through the triboelectricity signal generated by TENG, droplets oscillate (or move laterally) on the glass surface and condense due to the difference of triboelectric potential distribution. When the combined droplets are large enough (gravity is greater than resistance), the droplets will fall from the glass to realize the function of anti-fogging.

The access control system is the primary requirement for smart home security system. Recently, Qiu et al. proposed an innovative control disk interface based on self-powered TENG for smart home and access control applications [47]. As shown in Fig. 7d, eight control commands can be realized by encoding eight codes on the sensor through a 3-bit binary code. The smart home access control system is divided into outdoor modules (TENG control disk, control circuits, and an MCU for digits decoding) and indoor modules (the password recognition MCU and door lock control circuit). During daily use, after the owner wakes up the outdoor module from sleep mode by manually entering the password, the MCU will analyze the electrical signal collected by the TENG control panel. Subsequently, the analysis result will be transmitted to the indoor module via Bluetooth module. When the password is correct, the door will open. Through the application of IoTs in smart home based on TENG energy harvesting and sensing technology, it has attracted a lot of attention and presented huge market prospects from the aspects of smart floors, smart windows, HMIs, etc. With the development of TENG sensors, intelligent interaction in real-world and VR/AR environments plays an important role in HMI applications. Advanced technology approaches (e.g., big data and AI) are also further promoting the relevant applications of smart homes in the 5G era [38], which will be discussed in Sect. 8.

### Smart Transportation

Smart transportation and traffic systems are essential to human safety and convenience in modern urban life, including vehicles, trains, ships, roads, bridges, etc. All of them are required to be equipped with various sensors. Advanced mechanical energy harvesters play an important role in ensuring the effective operation of these sensors [118–120]. Typical applications are divided into three parts, (i) the monitoring of driver’s behavior, (ii) the monitoring of driving tools (such as cars and trains), and (iii) the road or bridge related to transportation safety.

**Fig. 8:**
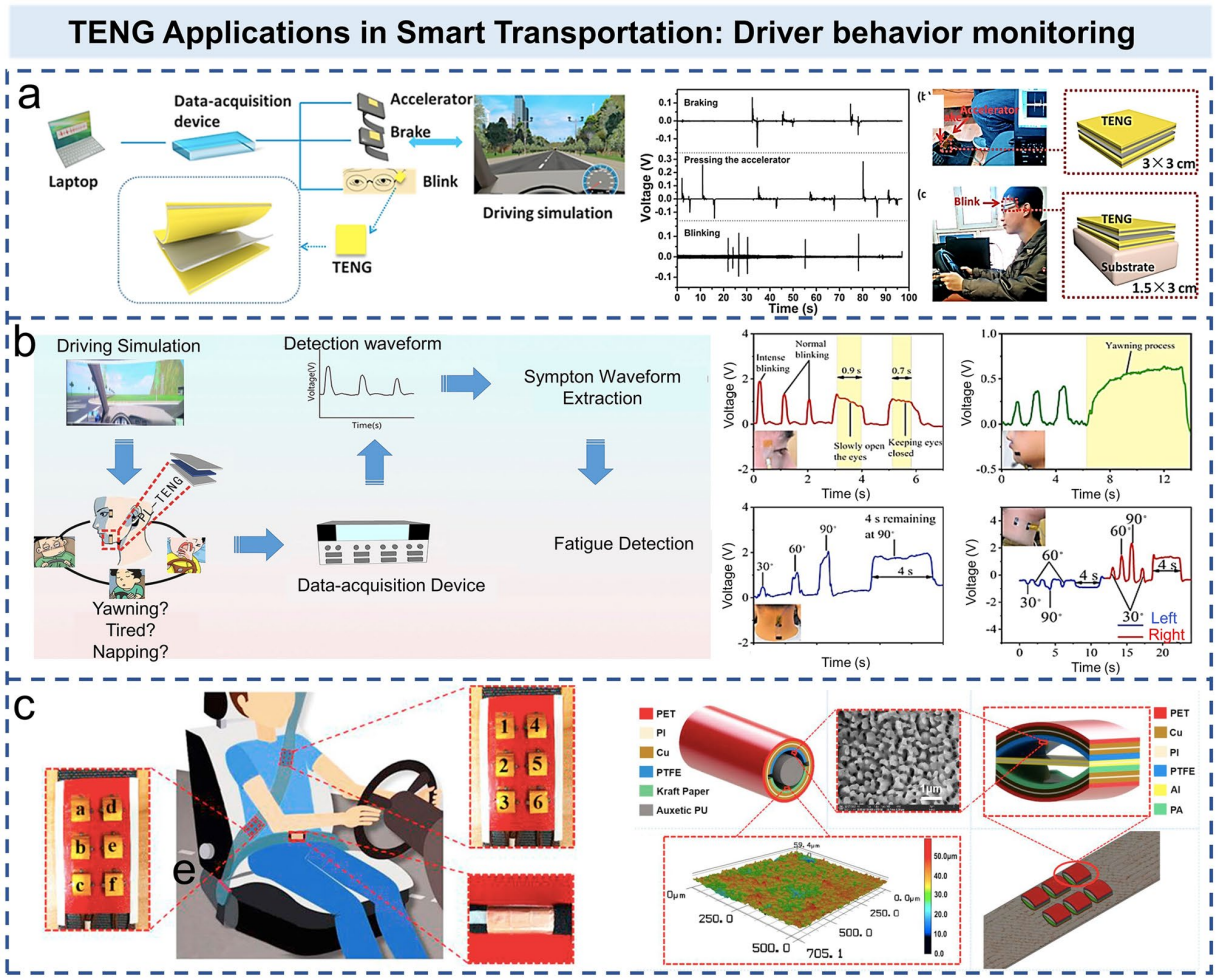
TENG applications in smart transportation: driver behavior monitoring. **a** Schematic illustration of self-powered triboelectric sensors for driver behavior monitoring and voltage signal acquisition [124]. Copyright 2018 Elsevier Ltd. **b** Schematic illustration of fatigue test and electrical signals of different actions (eyes closed, mouth open, nodding, and neck twisting) [125]. Copyright 2020 Elsevier Ltd. **c** Position diagram of the AS-TENG sensor arrays on the safety belt for monitoring the driver’s steering actions, and the structures of the APU-TENG and AS-TENG [126]. Copyright 2019 Elsevier Ltd

The increasing number of automobiles leads to a large number of traffic accidents every year. Driving fatigue and mental inattention are the two main causes of most traffic accidents. By monitoring the driving behavior of drivers, a timely warning can help to avoid the occurrence of man-made traffic accidents. Bright pupil effect based on near-infrared (NIR) illuminators is a widely used method to monitor driver’s behavior [121–123]. In recent years, TENG-based self-powered sensors have also received extensive attention in monitoring driving behavior. As shown in Fig. 8a, Meng et al. proposed a cost-effective sensing device made of aluminum foil and Kapton materials for driver behavior monitoring [124]. Based on the set-up common transport scenarios (including highway driving and passing through signalized or unaligned intersections), the electrical signals triggered by “blink,” “accelerator,” and “brake” actions are collected in real time, and valid information is extracted to determine whether drivers have fatigue driving and impulsive driving behavior. Compared with traditional NIR imagers, it has the characteristics of high sensitivity, high stability, and low cost. In addition to the blinking time and frequency, Lu et al. tested the mouth closure, neck rotation, and yawning as indicators of driver fatigue and distraction (Fig. 8b) [125]. Feng et al. reported a self-powered smart safety belt enabled by two TENGs to monitor driving status [126]. Xie et al. reported a novel swept TENG, which was composed of a pushrod, a housing, two flywheels, and a flywheel [127]. The habits of drivers were monitored by collecting the energy of flywheel motion randomly triggered by the pedal. After testing four volunteer drivers, the throttle signals were recorded when driving on the same road. The results show that different drivers have different driving habits, and long-term recording of this information is of great value to monitor drivers’ habits, further distinguishing them and predicting the risk of traffic accidents [127]. Xu et al. designed a smart system based on TENG, which was integrated into the vehicle steering wheel to record the rotation angle of the steering wheel of drivers [128]. The signal processing unit would analyze the recorded data to determine the warning threshold of each parameter, so as to evaluate the state of the drivers in real time. In Fig. 8c, an auxetic polyurethane–TENG is integrated into the horizontal strip of the seat belt to monitor the driver’s clearance to the seat belt, which is associated with sudden and aggressive deceleration. Two sensor arrays composed of arch-shaped TENG are installed at the shoulder and waist of the diagonal of the seat belt to monitor the steering direction and angle of drivers. These electrical signals are collected to analyze the state of drivers and prevent traffic accidents.

**Fig. 9:**
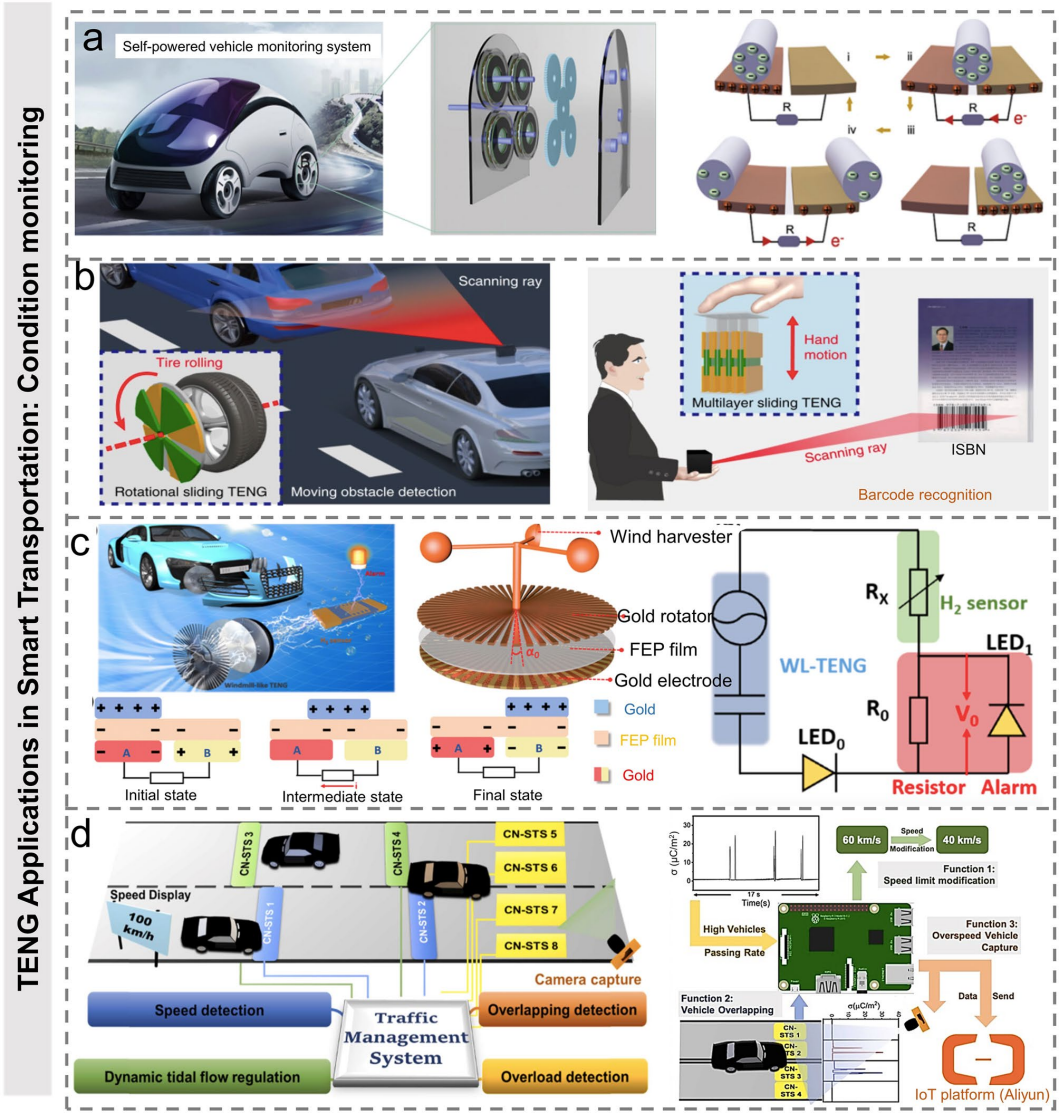
TENG applications in smart transportation: condition monitoring. **a** Working principle of self-powered vehicle monitoring system fixed on the main axis of automatic vehicles and BS-TENG rolling independent mode [129]. Copyright 2021 The Author(s). Published by Elsevier Ltd. **b** Schematic illustration of the TM-based portable scanner for book ISBN recognition by slow gesture recognition, and the TM-based scanning system for moving obstacle detection by low-speed tire rolling [50]. Copyright 2019, The Author(s), published by Springer Nature. **c** Schematic diagram of the self-powered hydrogen leakage sensing system, structure and working principle of WL-TENG, and equivalent circuit with divider resistor of the self-powered H_2_ sensor monitor system [49]. Copyright 2021 Elsevier Ltd. **d** Functional demonstrations of speed limit adjustment, vehicle overlapping detection, and speeding capture for the traffic management system realized by the CN-STS and real-time charge density output signals through Raspberry Pi [134]. Copyright 2021 Elsevier Ltd

Hardware monitoring on the vehicle itself is an important prerequisite for safe driving. Large-scale signal processing systems and sensor networks have been widely used for vehicle monitoring to ensure a safe and reliable driving environment. As shown in Fig. 9a, Yang et al. used 3D printing to fabricate a novel bearing structure TENG, which can be used as a rotating mechanical energy harvester and self-powered sensory system for high-speed sensing [129]. The self-powered vehicle sensing system is composed of a high-speed sensor and a seven-unit energy-harvesting network, which can monitor local temperature and vehicle speed in real time. Yang et al. proposed a triboelectric micromotor (TM) driven by ultra-low-frequency mechanical stimulation for mobile obstacle monitoring [50]. As shown in Fig. 9b, the authors demonstrated two information recognition scanning systems. One is a portable scanner based on TM, which generates electric energy through multilayer mechanical sliding generated by low frequency and drives a micromotor to generate scanning rays. The other is a moving obstacle detector based on TM, which drives the micromotor and scans the moving obstacles through the rotating TENG of normal tire rolling and uses the autonomous information recognition technology to detect the potential danger of the vehicle. As the condition of tires is a crucial parameter, Qian et al. have developed an on-vehicle magnetically TENG to harvest rotational energy from tires and serve as a direct power source for a tire pressure sensor to monitor the internal pressure of pneumatic tires [130]. Thus, it can effectively help to evaluate the state of the vehicle. The key component of the automobile braking system is the brake pad. With the operation of the braking system, the brake pads will inevitably wear. It is very important to understand the mechanical wear degree of brake pads in the braking system [131]. Kim et al. developed the geometric gradient contact layer assisted smart brake pad with the triboelectric effect-based design strategy (G-TBS) [132]. A physically asymmetric composite material was employed to get the spatially non-uniform electric potential distribution, which provided the method to naturally perceiving the level of the mechanical abrasion. Two types of friction materials (PDMS, PLA) were introduced into the brake pad system through geometric design. When the brake system started to operate, the wear ratio (WR) of the G-BTS would increase from 0%. The increase in WR was closely related to the total exposure area of PDMS and corresponding sensing signals could be spontaneously generated due to the contact electrification between the PDMS and PLA. Thus, the magnitude of the triboelectric signal generated from the brake system could directly indicate the abrasion level of the G-BTS. The proposed G-BTS realized the real-time wear monitoring in a self-powered manner.

In Fig. 9c, Jiang et al. showed a self-powered hydrogen sensor based on an impedance adjustable windmill-like TENG [49]. The self-powered H_2_ sensing system consists of three main parts: TENG, H_2_ sensor, and alarm LED. When the H_2_ concentration changes, the output signal will be influenced, which will lead to the brightness change of the alarm LED. With the development of new energy vehicles, real-time monitoring of hydrogen leakage is of great significance during vehicle operation. Moreover, in order to further promote the development of intelligent transportation systems, Tang et al. proposed a TENG-based wireless monitoring system to bicycles, electric bicycles, and motorcycles, which can monitor them illegally entering the sidewalk, speed measurement, and the driving direction on non-motorized lanes [133]. Yang et al. developed a self-powered triboelectric sensor made of electrospun composite nanofibers for intelligent transportation monitoring and management [134]. To meet the requirements of fast response and high sensitivity for the smart traffic management, the transferred charge density was adopted as the sensing signal which could perfectly record the subtle differences and was more suitable for dynamic traffic monitoring (compared to the voltage or current signal). The adoption of carbon nanotubes doped into PVDF nanofibers can greatly improve the electrical output performance and pressure sensitivity (0.0406 C m^−2^ kPa^−1^ at low pressure range and 0.0032 μC m^−2^ kPa^−1^ at higher pressure range). Notably, using charge amplifiers in power management to process the charge input signals, the vehicle signal and human walking signal can also be clearly distinguished. By connecting to cloud IoTs service, the functions of traffic flow management, overlapping and speeding vehicle capturing, and plate number recognition were realized by the self-powered TENG sensor arrays with a compensation circuit (Fig. 9d). In contrast to this, Shan et al. designed a novel inverting TENG to realize the conversion from DC to AC signal based on the coupling of triboelectrification and air breakdown [135]. The inverting-TENG exhibited unique characteristics that both the pulse width ratio and amplitude ratio of AC signals can be controlled by tuning the distributed width and electronegativity difference of two opposite materials. Benefiting from these characteristics, the authors designed a real-time computer-simulated displacement and direction controller for a car, which demonstrates its potential applications for real-time cars position monitoring and car parking management.

**Fig. 10:**
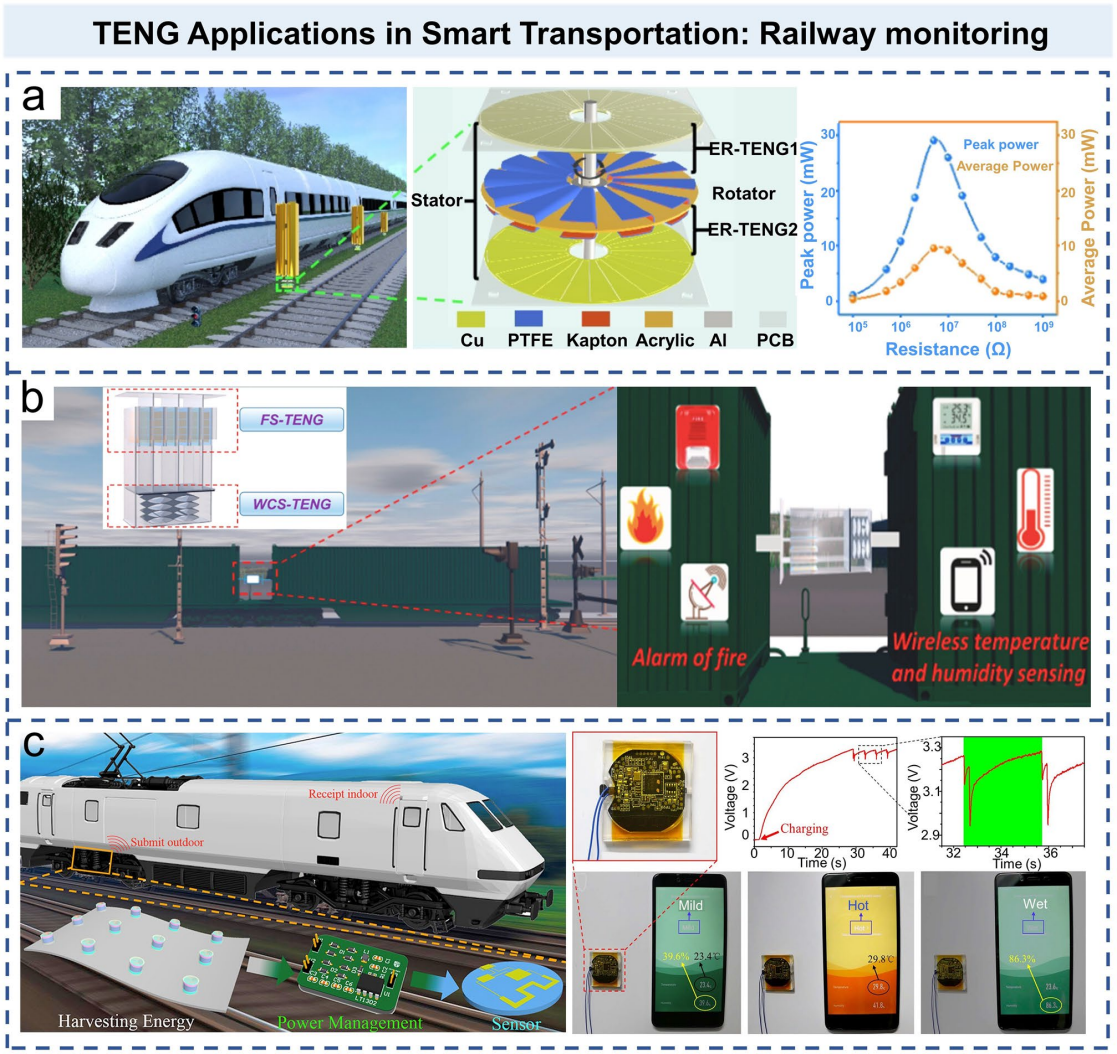
TENG applications in smart transportation: railway monitoring. **a** Structure design and output power of elastic rotation TENG [136]. Copyright 2021 American Chemical Society. **b** Multiple-mode TENG with charge excitation collects environmental mechanical energy for self-powered freight train monitoring [137]. Copyright 2021 Elsevier Ltd. **c** Self-powered wireless smart sensor based on magnetic levitation porous nanogenerator for a train monitoring system [138]. Copyright 2017 Elsevier Ltd

The trains and high-speed rails running on the track will generate enormous wind energy due to their fast speed. In order to collect this wasted wind energy, as shown in Fig. 10a, Zhang et al. fabricated an elastic rotary TENG (ER-TENG) to harvest the wind energy generated by high-speed trains and supply the power to relevant sensing devices [136]. The transferred charge (*Q*_SC_), short-circuit current (*I*_SC_), and open-circuit (*V*_OC_) obtained by ER-TENG are 0.9 μC, 120 μA, and 600 V, respectively. When the load resistance is 50 MΩ, the obtained peak power is 29.1 mW. Du et al. designed a multi-mode TENG (MM-TENG), which is composed of multilayer floating sliding components and multilayer corrugated contact-separation components [137]. It is used to harvest environmental mechanical energy at the junction of freight cars and supply power to the sensors for monitoring the train status (Fig. 10b). The multilayer waveform contact-separated TENG provides charge excitation to the entire TENG, which greatly improves the overall output performance of the MM-TENG. Similarly, Jin et al. developed a magnetic levitation porous nanogenerator (MPNG), which effectively combines TENG and EMG (Fig. 10c) [138]. The device is used to harvest the vibration energy of the train and continuously supply power to the wireless smart sensors. At 20 Hz, the peak *V*_OC_ of TENG is 43.8 V and the *I*_SC_ is 1.39 μA, and the maximum *V*_OC_ of EMG is 7.7 V and *I*_SC_ is 4.1 mA. By connecting with external resistors, TENG provides a peak power density of 0.34 mW g^−1^ at 50 MΩ, while EMG delivers a maximum power density of 0.12 mW g^−1^ at 700 Ω. MPNGs have the potential of wireless monitoring without an external power source, especially in the train monitoring systems.

**Fig. 11:**
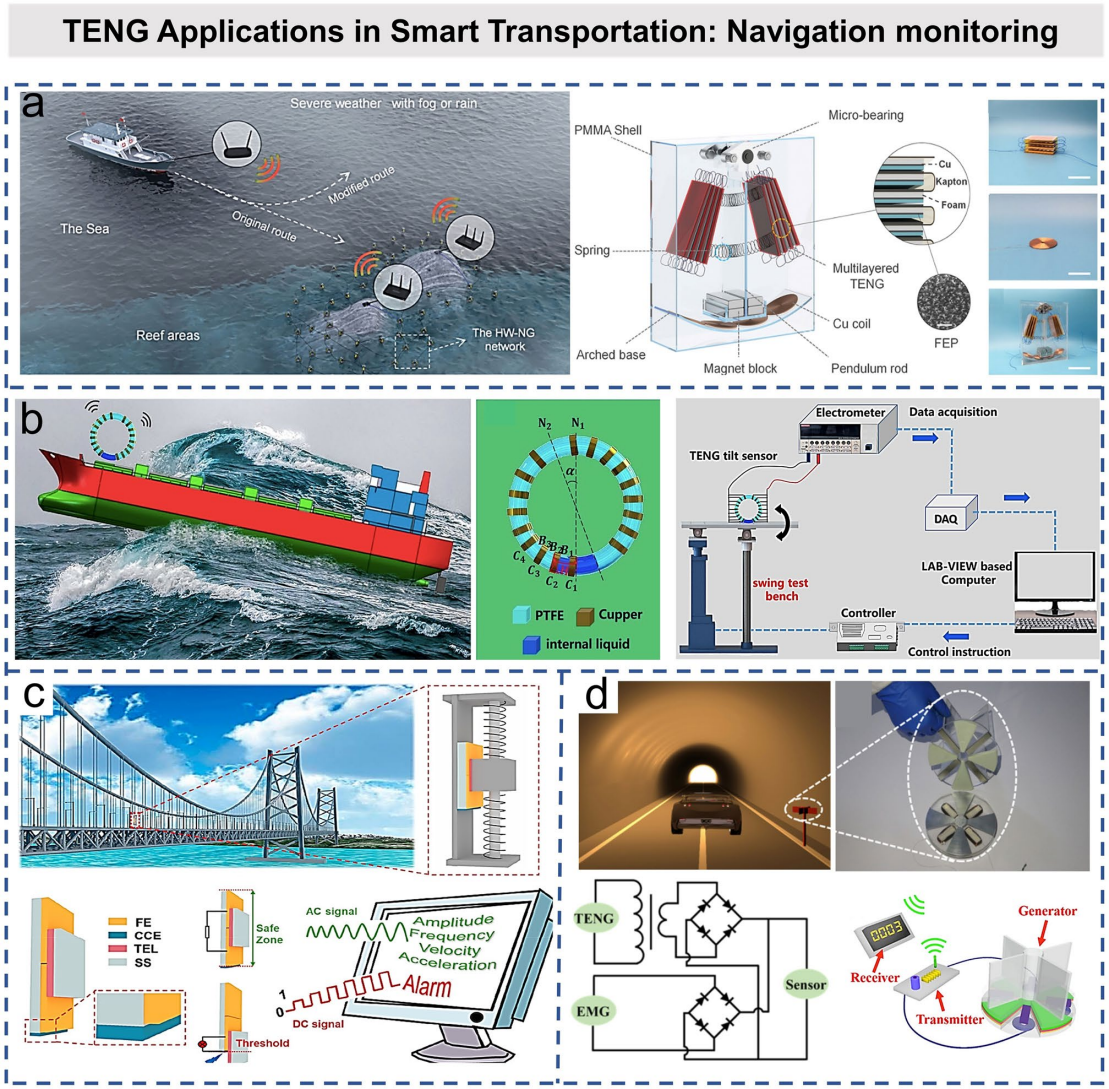
TENG applications in smart transportation: navigation monitoring. **a** Schematic of hybrid wave energy harvesting nanogenerator (HW-NG) network distributed in the waters adjacent to coral reefs, which is used to harvest wave energy, and the developed automatic RAW system for ocean navigation [141]. Copyright 2021 Wiley–VCH GmbH. **b** Schematic diagram of TENG tilt sensor applied to ship attitude sensing and experimental apparatus [143]. Copyright 2020 Elsevier B.V. **c** Schematic and experimental structure of a fully self-powered vibration monitoring sensor driven by AC/DC-TENG [145]. Copyright 2020 American Chemical Society. **d** Schematic diagram of the sensor working in the tunnel, and the enlarged view of the self-power sensing system [146]. Copyright 2016 American Chemical Society

Like cars and trains, ships are driven by large power sources, but the distributed sensors on-ship require continuous low-power driving. If these sensors can be driven by the harvested wave vibration energy, it will be a very attractive application. On this basis, Zhang et al. proposed a hollow-ball buoy-assisted triboelectric ocean-wave spectrum sensor (TOSS) [139]. The TOSS can monitor wave height (*H*), period (*T*), frequency (*f*), wave speed (*v*), wavelength (*L*), and wave steepness (*δ*) in real time. Due to the low cost and simple structure of sensors, large sensor arrays can be used to detect waves in a wide range of sea areas. Based on TENG, a magnetic flip-plate dual-function sensor (MFTDS) was developed to detect pneumatic flow and liquid level [140], which could detect the pneumatic flow rates ranging from 10 to 200 L min^−1^ and had a flow resolution of 2 L min^−1^. As shown in Fig. 11a, Ren et al. proposed a hybrid nanogenerator with TENG and EMG as power source, which harvested wave energy for long-distance (1.5 km) wireless transmission [141]. The integration of TENG and EMG for hybridized wave energy harvesting can combine their complementary advantages. Based on this, they developed a self-powered route avoidance warning system for navigation at sea to ensure the navigation safety of ships in all weather conditions. An et al. combined the advantages of sensors based on TENG and slug assemblies to construct self-powered slug mechatronics (BIM) panel [142]. With the help of magnetic floats, the BIM panel can be used as a reliable mechanical indicator to directly reflect the induced liquid level and flow characteristics through the visible color change of the flap and can also be used as an electronic sensor for automatic control and remote wireless monitoring to ensure the safe transportation of cruise ships. Figure 11b shows a robust and self-powered tilt sensor for ship attitude sensing [143]. Wang et al. designed an annular PTFE tube with copper electrodes segment disposed on the surface, and the internal liquid was encapsulated in PTFE tube without bubbles. Based on this, they developed a tilt monitoring system to indicate the ship’s attitude in real time. When the inclination occurs, the internal fluid (pure water) flows through different electrode segments with the LED lighting up to indicate corresponding angles. Then, the inclination direction of the ship can be judged by the positive and negative value of *V*_OC_/d*t*. When the inclination of the ship exceeds the dangerous value, the visualization system can also trigger the alarm device.

As aforementioned, monitoring the condition of bridges and tunnels is also important for smart transportation. Lin et al. proposed and implemented a self-powered, flexible, timbo-like triboelectric force and bending sensor, which could effectively monitor the rockfalls and landslides near roads [144]. In Fig. 11c, Li et al. introduced a vibration sensor system for real-time continuous detection of vibration characteristics through a dual-mode AC/DC-TENG [145]. This integrated TENG vibration monitoring sensor provides a versatile response at different amplitudes: Within the safe amplitude range, the AC-TENG only produces AC signal, which can be stored in the capacitors to transmit the signal to the remote monitoring system. Once the safety threshold is exceeded, it will generate a DC signal and alarm in real time. In the whole process, the real-time monitoring on the safety status of bridge vibration can be realized without an external power supply. For tunnels monitoring, Bian et al. fabricated a TENG tree to provide lighting in the long tunnel and measure the vehicle speed by harvesting the wind energy generated by vehicle movement. In Fig. 11d, Zhang et al. reported an active wireless sensor for monitoring the traffic flow, which was based on the hybrid nanogenerator of spinning disk TENG and EMG [146]. Similarly, the hybrid nanogenerators can efficiently harvest energy from the wind generated by moving vehicles in the tunnel, and the delivered electricity will trigger the counter via wireless transmitters to indicate the real-time traffic volume in the tunnel.

In the intelligent transportation applications, TENG-based self-powered sensors/systems can be placed on vehicles/trains/ships or roads/bridges/tunnels, playing an important role in safety protection and early warning [50, 129, 137, 138, 145–147]. In addition to harvesting wasted mechanical energy, the proposed TENG or hybrid nanogenerator can also be used as self-power transmitters to deliver important information on traffic conditions. Moreover, the wireless network between vehicles and roads can be powered by the nanogenerators and used to warn and guide the transportation in advance.

## TENG Applications in Emergency Monitoring and Early Warning

**Fig. 12:**
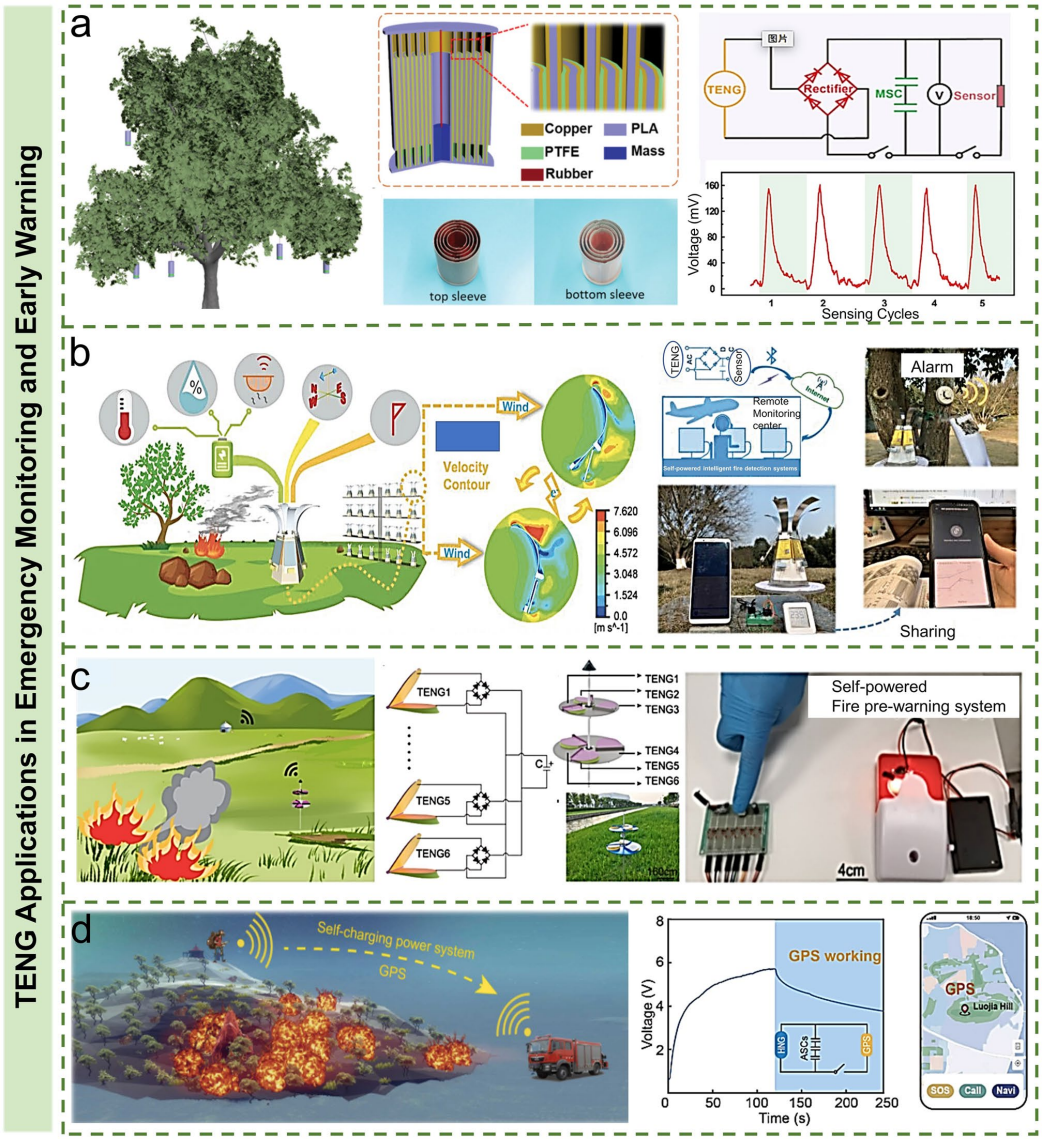
TENG applications in emergency monitoring and early warning. **a** Schematic illustration of the MC-TENG and self-powered fire alarm system for harvesting shaking kinetic energy of tree branches [148] Copyright 2020 WILEY–VCH Verlag GmbH & Co. KGaA, Weinheim. **b** Application prospect of the F-TENG in self-powered intelligent fire protection systems [149]. Copyright 2021 Wiley–VCH GmbH. **c** Application scene and the circuit schematic diagram of the self-powered wildfire pre-warning system [150]. Copyright 2021 Elsevier Ltd. **d** Prospect chart of an outdoor positioning device based on SCPS, voltage curves of the SCPS during the GPS charging and power supply. Schematic diagrams of the location information sent by the outdoor positioning device [151]. Copyright 2021 Elsevier Ltd

Emergency monitoring of sudden accidents is a kind of monitoring with a specific purpose. It requires the personnel for emergency management to arrive at the accident site as soon as possible; use small portable and rapid detection instruments or devices to judge the situation and hazards in the shortest time; and provide a scientific basis for the next decision. Intelligent emergency monitoring and early warning is an important part of emergency management. Timely and rapid feedback of information from the emergency source helps to reduce economic losses, casualties, etc. In recent years, the development of emergency management based on TENG has received extensive attention with their potential applications in fire monitoring, explosion warning, field survival/rescue, etc. [148–151]. In Fig. 12a, Pang et al. proposed a multilayer cylindrical TENG (MC-TENG) for harvesting the kinetic energy of tree branches, which could monitor environmental conditions and forest fires [148]. The MC-TENG consists of two major functional components. One is the top fixed sleeve suspended on the branches, and the other is the bottom sliding sleeve connected with the top sleeve by a highly stretchable rubber belt. These two components are combined into a spring–mass vibration structure. The authors integrated MC-TENG, fire sensor (commercial CO and temperature sensor), and micro-supercapacitor to build a self-powered forest fire alarm system, which can effectively avoid possible false alarms and improve the accuracy of the fire alarm.

Long-term disaster monitoring and early warning put forward higher demands and requirements on the relevant personnel. The arrival speed and working speed of emergency management personnel determine the efficiency of the follow-up rescue. Remote monitoring can realize real-time collection and rapid processing on the on-site monitored data, so as to reduce personnel consumption and overcome the limitations of long-term monitoring due to the limited labors. Zhang et al. illustrated a TENG based on fluid-induced vibration (FIV), which used Bluetooth wireless technology and mobile devices to construct a self-powered intelligent fire detection system (Fig. 12b) [149]. This system can respond to fire in time, and it can also be used to monitor the environmental information including the wind direction, speed, temperature, and humidity. Processing the above data, people can predict the speed and direction of fire spread, and even the fire risk. Gao et al. also reported a turbine disk TENG for wind energy harvesting and self-powered wildfire pre-warning (Fig. 12c) [150].

In the process of industrial production, granules are often transported through the pipelines, in which the particles collide with each other and are easily charged. Once they are discharged, it may cause fire and explosion and pose a threat to the safe production. According to the particle transport in pipes, Guang et al. developed a particle transport TENG (PT-TENG). The PT-TENG can be utilized to monitor the mass flow of particles and provide early warning to potential explosion without a power supply. In addition to explosion alarm, TENG-based applications also include powering the electronic devices/sensors related to first-aid and emergency management, which are of great significance for local survival or rescue. Cui et al. developed a hybrid nanogenerator by coupling EMG signals with TENG signals and constructed a self-powered locator for field survival/rescue by integrating with commercial electronics. To achieve good output performance and stable working state, Zhang et al. designed a high-efficiency self-charging power supply system (SCPS), which was composed of a rotary hybrid nanogenerator (R-HNG) with enhanced performance and a high-energy density asymmetric supercapacitor (ASC) for outdoor search and rescue (as shown in Fig. 12d) [151]. The output characteristics of the rotary TENG could be enhanced by adjusting the work function difference between the friction layer and electrode. The R-HNG composed of TENG and EMG exhibited outstanding energy scavenging ability since two nanogenerators could harvest more electrical energy from one mechanical movement. And the prepared ASC was used as an energy storage device to ensure smooth power supply from the SCPS to the homemade positioning device. The proposed research is expected to accelerate the development of SCPSs in the IoTs.

One of the key technologies for the development of IoTs is the sensing network, which can be considered as an integrated microelectromechanical system consisting of sensors, actuators, signal processing and control circuits, communication interfaces, and power supply components [152–156]. The goal is to integrate the function of acquisition, processing, and execution of information together to construct the micro-system with multi-functionality and significantly improve the level of automation, intelligence, and reliability. As is known, various sensors are the most important elements in IoTs, which will generate a large amount of sensory data during their operation. Efficiently utilizing/analyzing the data assisted with AI (especially machine learning with a strong data analysis capability) can deliver timely analysis and feedback. Accordingly, the integration of AI and the IoTs can promote better and more rapid development of the era of IoTs [157]. In the previous sections, we summarized the applications of TENG-based sensors in smart agriculture, smart industries, smart cities, emergency monitoring. These efforts will be more meaningful with more intelligent sensors capable of capturing, analyzing, and feedbacking the key information. Therefore, in the following section, we focus on the integration of intelligent sensing applications in IoTs aided by AI technology to better reveal the future application prospects.

## TENG Combined with Artificial Intelligence Technology for IoTs

In the era of 5G and IoTs, the HMIs provide humans with more intuitive interactions with the digital world and have flourished in the past few years. HMIs have gradually evolved from traditional computer peripherals (such as keyboards, mice, and joysticks) to more intuitive interfaces that can directly capture the human raw signals (e.g., voice and basic body motions) [158–160] and provide users with more intuitive and easier interaction with computers and intelligent robots in agriculture, industrial automation, smart city, VR game control, digital twins, etc. [38, 39, 161–163]. The integration of emerging AI and human–computer interaction technology has spawned a new field of intelligent systems, which can be detected, analyzed, and decided by ML-assisted algorithms [164, 165]. Traditional signal analysis methods can help to manually extract basic features from the sensory signals [166, 167]. AI techniques can not only assist sensors in detecting more complex and diverse sensor signals, but also automatically extract the special features from sensor signals that represent the internal relationship of data sets [168, 169]. Due to the powerful feature extraction ability of ML, more comprehensive/detailed sensory information can be utilized to achieve diverse applications (e.g., gesture/pose estimation, speech recognition, object recognition) [26, 30, 170], serving more advanced HMI system adaptable to the construction of more intelligent sensor in IoTs. In this section, various HMIs based on TENG assisted with ML methods, AR/VR applications, and digital twins are summarized to promote the construction of IoTs.

### AI Applications of TENG with Machine Learning

**Fig. 13:**
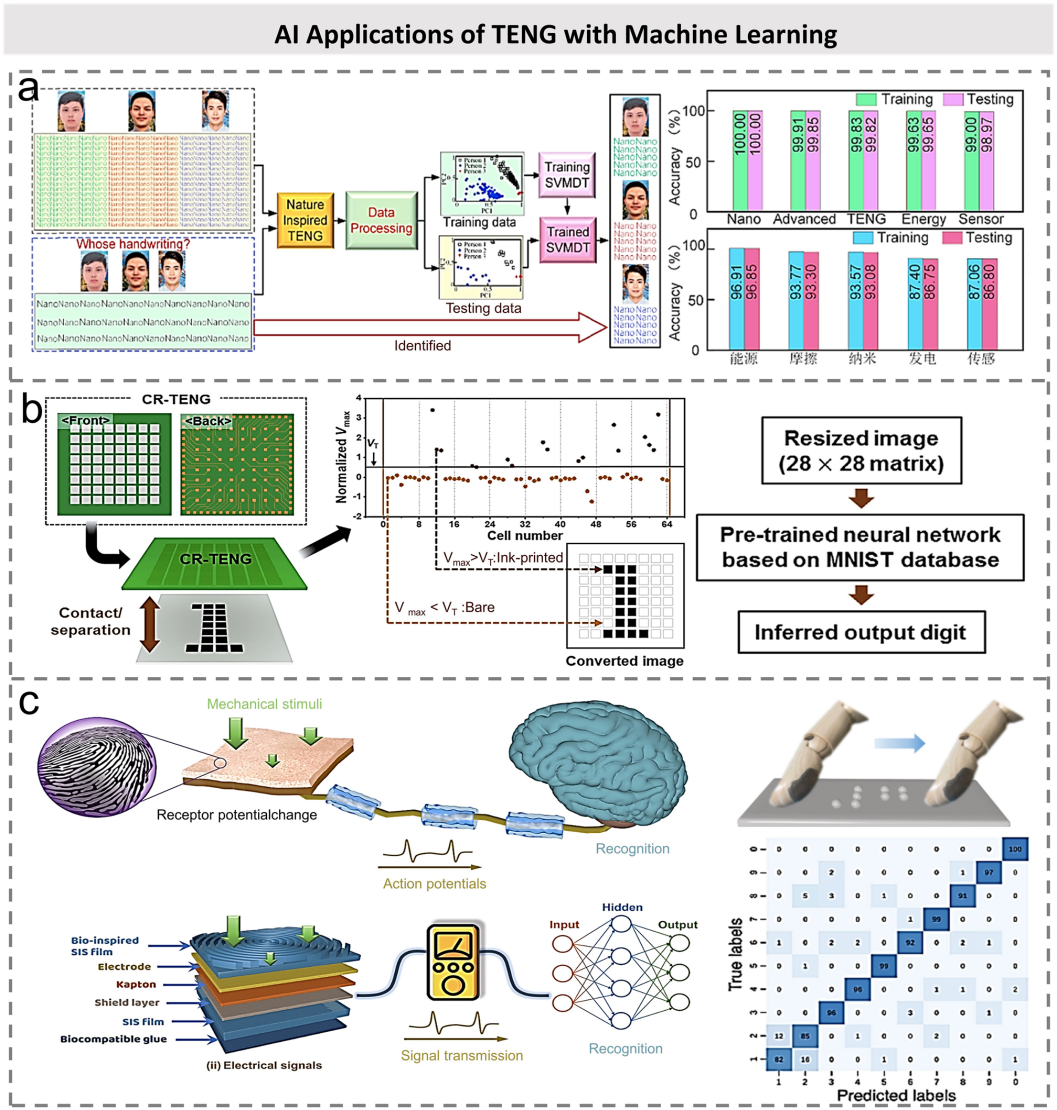
AI applications of TENG with machine learning. **a** Combining leaf-inspired TENG and machine learning method, the process of handwriting recognition is demonstrated [37]. Copyright 2020 Elsevier Ltd. **b** Scanning process based on the CR-TENG and the flowchart of the character recognition process of the system [174]. Copyright 2020 Elsevier Ltd. **c** Design of biometric tactile system based on fingerprint-sensing electronic skin (FE-skin) and artificial neural network [29]. Copyright 2021 Elsevier Ltd

Recently, a large number of ML-assisted AI applications based on TENG were successfully developed based on support vector machine (SVM), neural network (NN), deep learning (DL), etc., where the subtle features hidden in the triboelectric waveshape have been proven to effectively enhance the recognition ability of intelligent sensory system (Table 3) [29, 35, 171–173]. Handwritten signatures are widely used in daily life, and the determination of signature authenticity is particularly important. However, the main challenge of handwriting recognition is to obtain comprehensive information features of the signature. Since TENG is responsive to external trigger forces, it can be used to record the signals and unique characteristics of individual’s writing process. On this basis, Zhang et al. proposed a multilingual-handwriting self-powered recognition system to implement handwriting recognition by combining a handwriting pad with ML methods [37]. The traditional offline handwriting recognition and identification method is to present the complete writing information in the form of images and then scan the handwriting data on paper and convert it into digital format, which is time-consuming and costly. Compared with traditional recording methods, handwritten data can be cheaply acquired and saved as digital signals assisted with TENGs due to the abundant material selection, low cost, and easy fabrication. Because of the difference in writing pressure, speed, acceleration, etc., the handwriting signal presents unique characteristics. As shown in Fig. 13a, for the multi-classification problem, the multi-classification model relying on SVM-based decision tree (SVMDT) was employed as the preferred classifier. The tenfold cross-check method was used for training and testing the SVDMT to validate the effectiveness of the handwriting recognition system. The results in Fig. 13a clearly showed that all test data of the English word “nano” were correctly identified. Tcho et al. developed the character recognition TENGs (CR-TENGs) using neural networks, and the schematic illustration of the overall character recognition operation is shown in Fig. 13b [174]. The CR-TENGs have 64 square cells. When there is digital input, digital images of paper patterns can be obtained through the different output voltages from each cell. The authors then used the neural network to infer the digital type of the converted images. The neural network is pre-trained using the modified national institute of standards and technology (MNIST) database. By optimizing the reference voltage and proportion ratio, a high recognition rate is achieved. In addition, TENG-based sensors can also be used to recognize microtextures with the help of the ML method. Inspired by human tactile perception system, Zhao et al. proposed a fingerprint-inspired electronic skin (FE-skin) based on SE mode TENG to detect the fine texture [29]. In the process of touch, because the contact area and pressure between the FE-skin and the object change with time, the surface texture is mapped according to the varied output voltage in real time. The fingerprint structure of the friction layer can effectively increase the frequency range and intensity of the tactile response. Furthermore, artificial neural networks are used to classify and identify the collected signals. Through this combination of software and hardware, the authors constructed the bionic tactile perception system with fine texture recognition ability. As shown in Fig. 13c, after recognizing 10 kinds of ordered texture Braille characters, the accuracy of 1,000 training sets and 200 verification sets reached 93.7% and 92.5%, respectively.

**Table 3:** Machine learning-assisted TENG sensors for AI applications

Refs.	Signal magnitude	Algorithms, models	Accuracy rates (%)	Applications
Zhao et al. [29]		ANN	93.33	Texture recognition
Syu et al. [35]	nA	LSTM	82.3	Gesture identification
Zhang et al. [37]	nA	SVMDT	99.66	Handwriting recognition
Zhang et al. [171]	V	RF	92	Action detection
Yun et al. [172]	V	CNN	93.6	Touch pad
Maharjan et al. [173]	V	ANN	99	User identification
Ticho et al. [174]	V	DNN	89	Character recognition
Zhao et al. [175]	μA	DBN	98.01	Intelligent keyboard
Wu et al. [177]	V	SVM	98.7	Keystroke identification
Zhang et al. [178]	V	CNN	97.5	AI-toilet

**Fig. 14:**
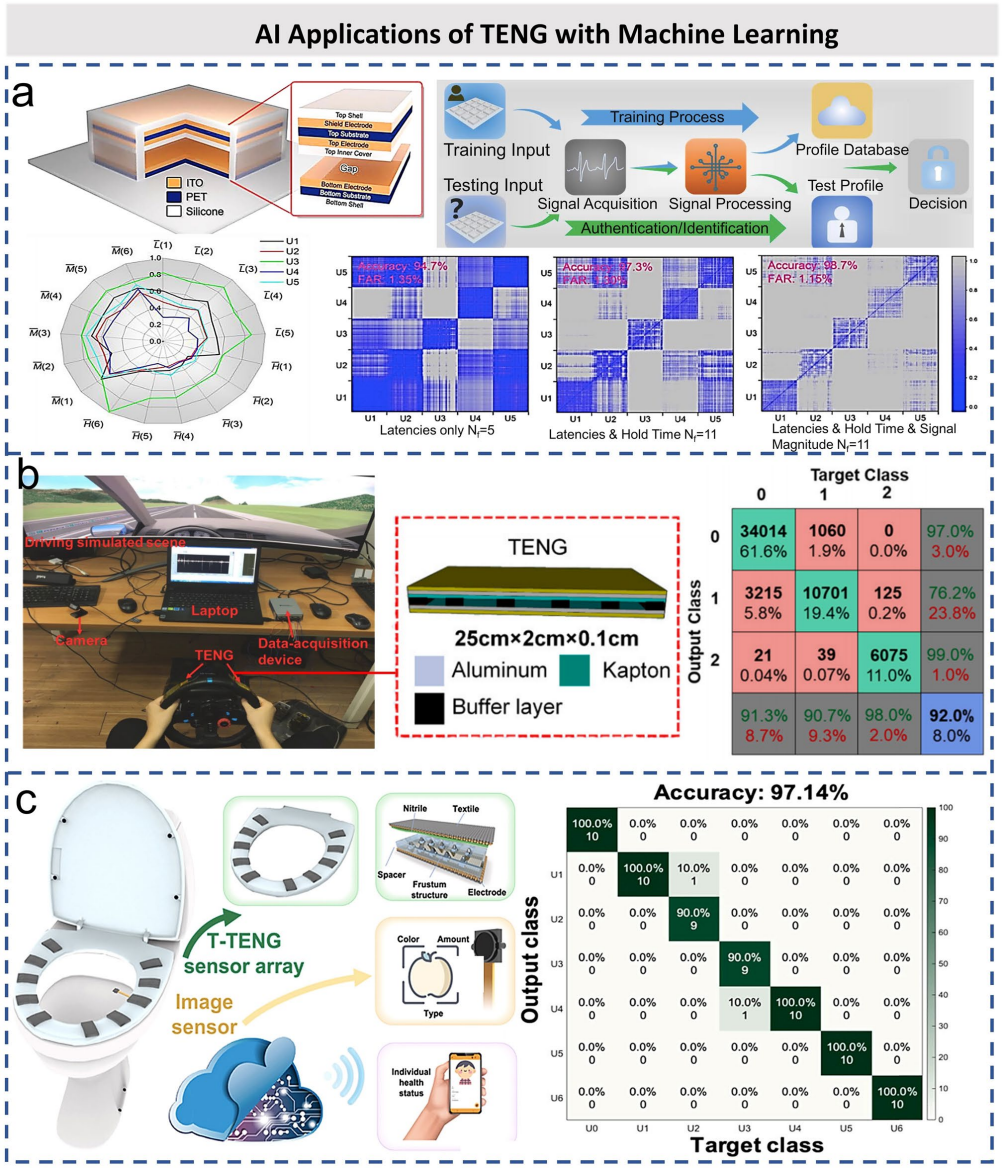
AI applications of TENG with machine learning. **a** Schematic and exploded view of a single triboelectric key. The system overview includes the training process and the authentication/identification process, and the difference matrix between user inputs of different feature types combinations [177]. Copyright 2018 Elsevier Ltd. **b** Experimental platform and the framework of the proposed method [171]. Copyright 2020 Elsevier Ltd. **c** Artificial intelligence toilet (AI-Toilet) for integrated health monitoring system and t-SNE diagram of sitting posture dataset recorded from the pressure sensor array [178]. Copyright 2021 Elsevier Ltd

In terms of the network security, the authentication system based on keystroke dynamics has been proved to be an effective method, which can improve the security level according to the human typing attributes of noninvasive monitoring features [167, 173, 175, 176]. As shown in Fig. 14a, Wu et al. developed a smart keyboard based on TENG for dynamic monitoring of keystrokes [177]. The multi-channel keyboard array consists of 16 highly flexible and malleable silicon-based keys, and each key consists of a CE mode TENG and a shield electrode. The TENG is used to capture the electrical signal of the typing behavior, while the shielding electrode helps to reduce the environmental interference. Different voltage signals can be extracted from the keystroke-related features, such as typing delay, hold time, and voltage signal amplitude. After the principal component analysis (PCA), the multi-class SVM classifier is used for authentication and identification. The accuracy is as high as 98.7%, which shows that the authentication system based on keystroke dynamics is feasible for practical applications. Besides, Maharjan et al. proposed a keystroke biometric authentication system based on hybrid electromagnetic–triboelectric sensors assisted by artificial neural network (ANN) [173]. DL (as a subfield of ML) can provide an effective way to automatically learning the representative features from the collected raw signals by training end-to-end neural network. By acquiring the keystroke information of users, the DL neural network continuously learns and adapts to the keystroke behavior of users, such as typing force, hold time, flight time, and interval, thus providing dual security for user authentication. Compared with other ML platforms, the neural network learns and adapts in the system to provide more sophisticated personal security systems. This battery-free keystroke sensing system and the software system based on neural network can accurately differentiate and verify different users with 99% accuracy throughout their individual typing habits.

In addition to the keystroke dynamics, driving behavior-based monitoring and recognition is another promising technology in biometric authentication applications. With the help of AI technology, the driver’s steering action can be recognized by analyzing the driver’s steering operation to establish a steering wheel motion model. Zhang et al. investigated and verified the possibility of using TENG to detect the steering wheel driving behavior [171]. To compare the response speed of different sensors (driving simulator, camera, and TENG) and evaluate the accuracy of steering action detected by TENG, two experiments based on driving simulators are designed, and the experimental framework is shown in Fig. 14b. At the same time, a supervised ML algorithm is developed to detect the driver’s steering actions, and the results show that TENG has the fastest response speed statistically. The algorithm trained with the random forest (RF) classifier has the highest accuracy of 92.0% in the test dataset, which demonstrates the potential application of TENG as the sensors for drivers’ steering actions.

Recently, the combination of TENG pressure sensor and DL method has been proposed for biometrics recognition of artificial intelligence of toilet (AI-toilet). Zhang et al. demonstrated an AI-toilet system equipped with multiple functions for an integrated health monitoring system [178]. Figure 14c shows the schematics of the AI-toilet, and it is configured with an array of triboelectric pressure sensors for biometric recognition and a commercial image sensor for medical monitoring. Ten textile-based TENG sensors are placed on the toilet seat to detect the corresponding pressure changes when the user sits down. The design of the friction layer with spacers and frustum structure can improve the sensitivity and detection range. AI-toilet has the advantages of high privacy, low cost, and easy preparation. With the help of DL method, the biometrics information of 6 users sitting on the toilet seat was successfully identified with an accuracy of 90%. The AI-toilet can not only process the data collected from the hardware side, but also process the multimodal data interpreted from the software side. Finally, it can be combined with IoTs to embed the AI-IoT system into smart home.

### AI Application of TENG with AR/VR Technology

**Fig. 15:**
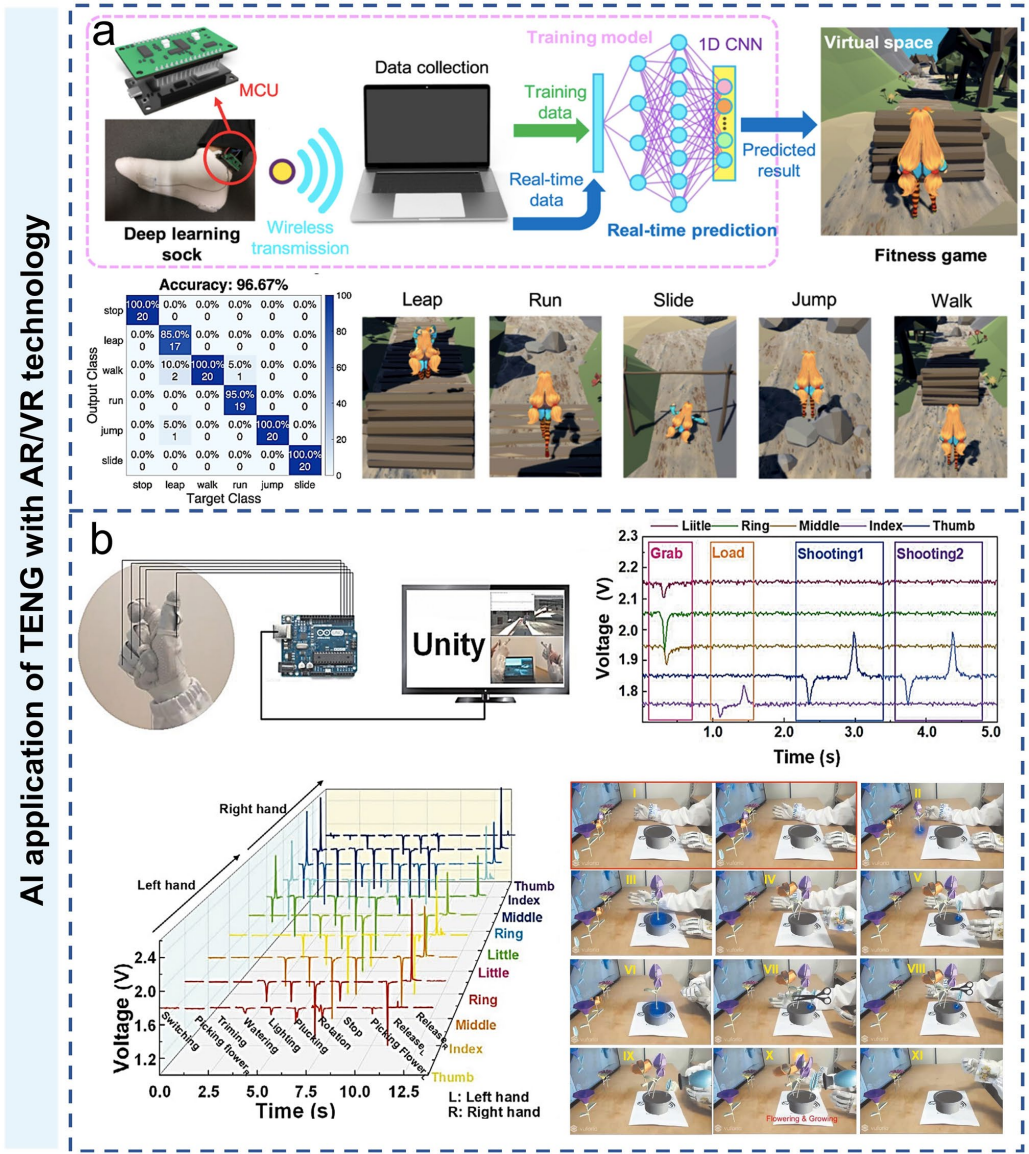
AI applications of TENG with machine learning. **a** Process from sensory information collection to the real-time prediction in VR fitness games and the confusion map of deep learning results [32]. Copyright 2020, Copyright The Author(s), Published by Springer Nature. **b** Demonstration of shooting game, which is based on the amplitude of output signals. And the AR demonstration of flower arrangement based on machine learning for complex gesture recognition [48]. Copyright 2020 The Authors. Published by WILEY–VCH Verlag GmbH & Co. KGaA, Weinheim

With the commercialization of AR and VR technology, the virtual world has become a reality. The rapid development of AR and VR technology has laid the foundation for diversified applications in social media, industrial production simulation, surgical training, games, and so on. Similar to the interaction between human and virtual world, AR and VR systems rely on HMI sensors to interact with the virtual world. In addition to audio and visual feedback, this immersive experience may involve other types of tactile feedback. Therefore, the wearable sensors based on TENG have the advantages of ultra-thin, ultra-soft, conformal, and imperceptible, which help to provide a more extreme experience for AR/VR technology [179–183]. The identity recognition based on gait analysis is also a promising biometric technology [166, 184]. On the one hand, with the help of AI, information about user identity and real-time activities can be transmitted by analyzing the physical signals obtained from the sensing systems of the floor or socks. On the other hand, the collected physical signals can be mapped into virtual space, which can be used to build a digital human body system for motion monitoring, healthcare, identification, and future smart home applications. Lee’s group has proposed a textile-based TENG sock to assist DL techniques for gait analysis [32]. As shown in Fig. 15a, this TENG consists of the silicone rubber film with patterned frustum structures, the nitrile film, and conductive textiles, which are used as negative triboelectric layer, positive triboelectric layer, and output electrodes, respectively. The textile-based TENG integrated into the smart sock for gait monitoring shows high pressure sensitivity of 0.4 V kPa^−1^ and large sensing range of > 200 kPa. Using the optimized four-layer one-dimensional (1D) convolutional neural network (CNN) model to automatically extract features from the walking spectrum, the recognition accuracy of 13 participants was 93.54%, and five different human activities were detected simultaneously with an accuracy of 96.67%. In a practical scenario, the authors demonstrated a VR fitness game with smart socks as the control interface, which had great prospects in the development of digital people. After this work, they further developed a textile-based triboelectric sensory system for gait analysis and waist motion capture. Compared with their previous work, it added robotic assistance for lower limb and low back rehabilitation [185]. They integrated smart insoles and harnesses into a lower extremity rehabilitation robot, demonstrating the system’s promise for user identification, motion monitoring, robot manipulation, and game-enhanced training. In addition to smart socks and insoles in the wearable field, Lee’s group also proposed a smart glove assisted with ML method, which uses self-powered conductive and superhydrophobic triboelectric textile for the gesture recognition in VR/AR applications [48]. As shown in Fig. 15b, the pristine textile is modified to be superhydrophobic by using a facile, scalable, and cost-effective coating method. On the one hand, superhydrophobic textiles are used to harvest biomechanical energy from human motion and as active sensors to monitor human motion. On the other hand, the integration of superhydrophobic textiles, the use of glove-based HMI, and the training of finger motion signals by ML are synergetic to realize complex gesture recognition. Due to the superhydrophobic capability of the device, the recognition accuracy under sweat conditions can still be maintained at 96.9%. Toward practical applications, the author demonstrated gesture recognition based on glove-style HMIs, which can realize 3D VR/AR scenarios of shooting, baseball pitching, and flower arrangement.

### AI application of TENG with Digital Twin Technology

Digital twin is a technology to make full use of physical model/signal update/operation history and integrate multi-disciplinary/multi-physical/multi-scale/multi-probability simulation process to complete the mapping in virtual space or Meta universe, which is possible to reflect the full life of corresponding entity equipment cycle process. In actual application scenarios, the digital twin system needs to integrate AI, ML, neural networks, and other methods to continuously learn and update the input information and adjust relevant operation mode. The combination of the digital twin and other related technologies (such as the IoTs) has provided favorable cyber-physical interaction and data integration. Recently, Lee’s group introduced several digital twin applications based on TENG, involving smart homes, intelligent manufacturing, virtual shop, etc. [33, 186, 187]

**Fig. 16:**
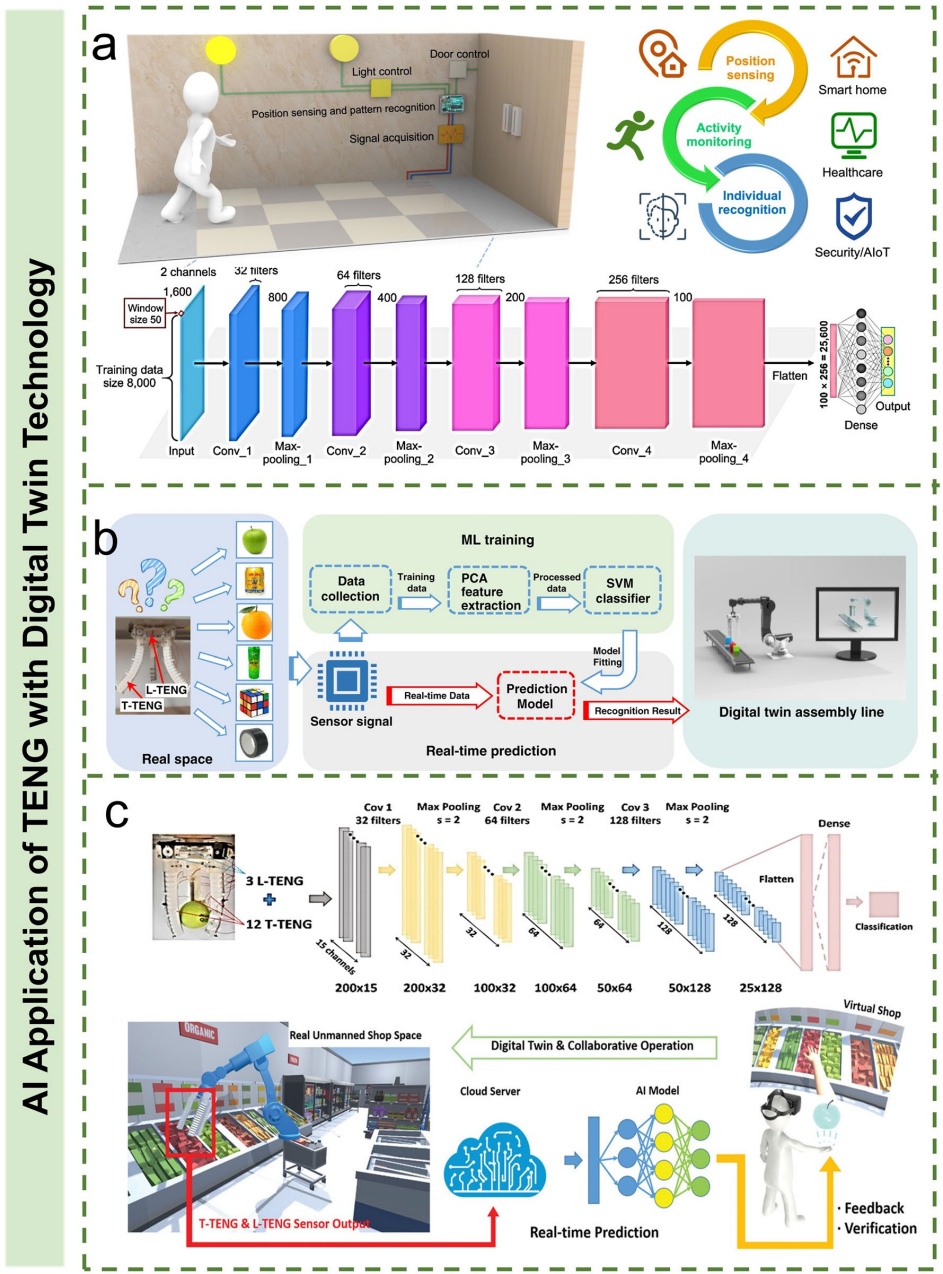
AI application of TENG with digital twin technology. **a** Smart floor monitoring system based on the deep learning-enabled smart mats (DLES-mats) [186]. Copyright 2020, The Author(s), Published by Springer Nature. **b** Schematic of the low-cost TENG for soft gripper and its digital twin applications. The process flows from sensory information collection to ML training and real-time prediction in digital twin system [33]. Copyright 2020, The Author(s), Published by Springer Nature. **c** System architecture of the digital-twin-based virtual shop and ML-enabled automatic grasped objects recognition system [187]. 2021 Copyright The Authors. Advanced Science published by Wiley–VCH GmbH

As shown in Fig. 16a, Shi et al. developed DL-enabled smart mats based on the triboelectric mechanism to realize an intelligent, low-cost, and highly scalable floor monitoring system [186]. The system was achieved by integrating the triboelectric floor mat array (with minimum electrode layout) with advanced DL-based data analysis. When the user walks through, the smart mat can obtain the differential signals. Through the integrated data analysis based on DL, the CNN model can be used to extract the identity information associated with walking gait patterns from the output signals. Based on this, they demonstrated an intelligent floor monitoring system that enabled real-time position sensing and recognition. The positioning information of each step can be used to control the light at designated position. Analyzing the complete walking signal can help to confirm whether the person is an effective user of the room, thereby automatically controlling the access control.

One of the digital twin applications in intelligent manufacturing is the classification of components in the workshop and the assembly of complex products. In the sorting lines of unmanned factories, sensors are built into the manipulators to identify the shape, size, and type of components. TENG-based sensors are more compatible with soft robots because the Young’s modulus of soft materials typically used in TENG sensors is at the same level with silicone rubber and thermoplastic polyurethane rubber (TPU) [48, 188, 189]. On this basis, Jin et al. reported a three-actuator soft gripper integrated with TENG sensors for digital twin applications [33]. TENG sensors are used to acquire signals and analyze data assisted with ML technology. It is successfully proved that the gripper perceives the grasping state and realizes object recognition. The smart gripper can successfully sense and recognize various objects with an accuracy of 97.1%, which can be further improved to 98.1% by increasing the number of sensor channels from 6 to 15. As shown in Fig. 16b, the scenes of digital twin are then established to replicate the digital information of the above manipulation in VR environment. By realizing real-time object recognition in a replicated virtual environment, digital twins can be further applied to the production control management of next-generation smart industry [187]. Another important scenario for digital twin applications is unmanned stores. Based on the enhanced soft manipulator, Lee’s team integrated PVDF pyroelectric sensors to achieve more complex sensing functions. To show the potential of the proposed intelligent manipulator for future online shopping and unmanned shop applications, they proposed a virtual shop system based on the digital twin model. As shown in Fig. 16c, through this system, users can select items in the digital twin virtual store, and the smart manipulator in the real unmanned store will simultaneously make corresponding actions based on the signals collected by the TENG sensors.

In the future, the combination of AI technology and IoTs technology will bring people a new living, working, and manufacturing environment. Imaging the future prospect under 5G and IoT infrastructure by utilizing the self-powered sensory interaction system (with the features of facile design, low cost, and high compatibility, etc.) together with AI techniques, a smarter society can be established toward intelligent industrial and automation, smart city modernization, smart agricultural mechanization, and normalization of emergency monitoring.

## Summary and Perspective

With a wide range of material choices and diverse structural designs, the smart sensing technology based on TENG has been widely studied because of its great potential in the construction of IoTs-related smart applications in this 5G era. In this review, we systematically summarize the application progress of TENG in multi-discipline application scenarios of IoTs, such as smart agriculture, smart industries, smart cities, emergency monitoring, and AI applications assisted by ML method. With the assistance of ML, AI technology has been introduced into the IoTs, which provides a promising research direction for the development of IoT technology.

**Fig. 17:**
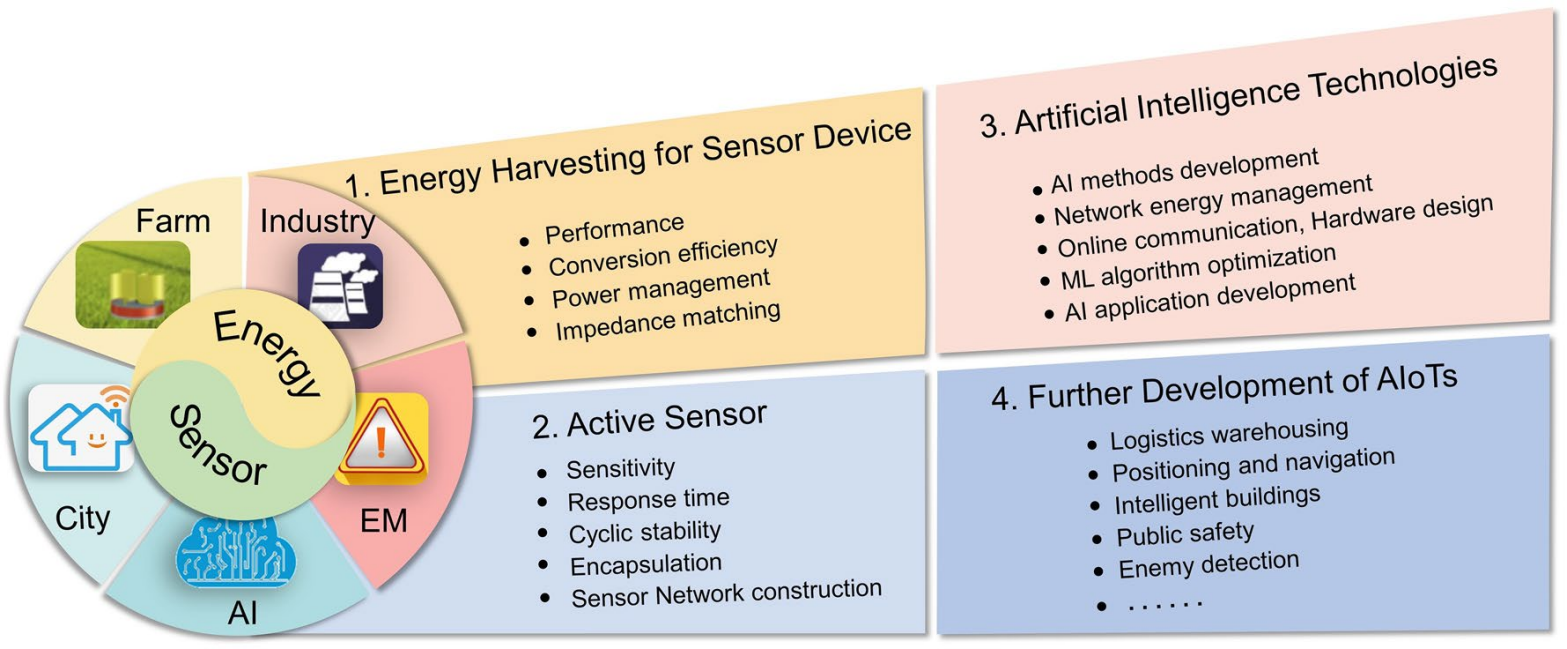
Challenges and perspectives of the IoTs application based on TENG sensors

In the future, the gradual promotion of IoT technology will greatly improve people’s lifestyles. Although the applications based on TENG (whether for energy harvesting or self-powered sensing) have made significant progress in the past period, there are still challenges to be solved in the practical application of TENG, such as power management and energy storage, service life, packaging technology, and large-scale sensor integration. The further development of the smart sensing applications of IoTs based on TENG needs to be considered from the following aspects (Fig. 17).

1. Whether in industry, agriculture, or smart city sensing applications, sensors mainly have two working modes. First, the device itself plays the role of energy harvesting. The AC signal is converted into DC, which can be directly used through the power management circuit to provide the required energy for subsequent commercial sensors [190, 191]. Paired power management is quite necessary for the application of TENGs because the harvested energy from surroundings is time-dependent, unstable, and susceptive to environmental changes. Meanwhile, corresponding energy conversion efficiency (an important technical index in energy harvesting systems) needs to be elaborately considered due to the energy consumption by some active electronic components during the AC/DC conversion [23, 192, 193]. Compared with common energy-harvesting and energy storage devices (e.g., supercapacitors, lithium-ion, batteries, solar cells, and thermoelectric cells [194–199]), the output characteristics of TENGs in high-voltage and low-current characteristics lead to an impedance mismatch between TENG and the power management circuit. Therefore, the transformers are often used to regulate the output performance and impedance of TENGs. However, because the harvested ambient energy is commonly low-frequency mechanical energy, the power management process using conventional transformers may also incur considerable power losses. The proper power management and energy storage methods need to be adopted between generator units and energy storage units to maximize output current and reduce power loss. In addition, some recently developed methods are also very effective to directly improve the output performance of TENGs at source (e.g., material optimization, surface treatment, pumping, and self-excitation). Furthermore, in order to enable the TENG network to harvest mechanical energy at large scales, the interconnection strategy among TENGs is essential.
2. Another typical application of TENG is to use it as an active self-powered sensor, in which the generated electrical signal is a direct response to the external environment. In this case, it requires the TENG active sensors with good sensitivity, fast response time, and wide detection range. Therefore, developing sophisticated structural design and effective working mode is particularly important. In practical application, the performance of TENG-based self-powered sensor may degrade to reduce the detection accuracy and service life. Therefore, the packaging of the self-powered device/system is also important. Researchers should make greater efforts in packaging materials and packaging technology to improve the stability and durability of the sensors. For example, researchers can select materials with excellent elasticity and mechanical properties (such as PDMS) to encapsulate the sensory devices to enhance durability or select appropriate encapsulation materials according to application scenarios (e.g., considering the biocompatibility of materials in the process of intelligent medical application). For multiple sensory devices, under the framework of IoTs, a large number of sensors need to be deployed for real-time sensing and data management/analysis. Therefore, it is very necessary to develop the integration of other modules in self-powered sensory systems to achieve favorable operation of the whole wireless sensory nodes (including the supporting components).
3. The combination of AI technology with IoTs technology will bring a new living, working, and manufacturing environment. In the construction of IoTs, the development of intelligent systems is the core issue. The overall architecture of IoTs intelligent system is composed of three layers: data collection layer, data communication layer, and data processing and analysis layer. In the self-powered IoTs, a large number of sensory nodes based on TENGs act as the data collection layer. Wireless networks are typical data communication layers. The cloud platform is used for data processing and analysis, and ML technology is a typical AI technology widely used in data processing [200]. The future research on AI-related smart in IoTs sensing application of TENG should be considered from the following aspects. First, people need to develop more advanced TENG sensors with embedded AI technology. When large numbers of TENG sensors are used as data sources for AI, the random characteristics of ambient mechanical energy (or information) may raise unavoidable problems for AI processing. For example, when the mechanical energy in the environment is insufficient, the obtained data by TENG sensor are random and the accuracy is low. AI technology needs to improve the incomplete information and improve the accuracy of information by learning the characteristics of complete information. Second, necessary power management for TENG sensory networks will also be a new research direction for the AI-related applications, which is helpful to solve the power consumption problem of traditional sensory systems. Third, most of the AI applications based on TENGs are in the early stage. For example, the data collection and data analysis in the process of machine learning often use offline analysis methods. The development of the auxiliary circuits between the sensor and the computer is lack of investigation, which limits the communication and practical application of the TENG-based IoTs sensory system. Therefore, in addition to the continuous development on TENG-based sensors, the development and design of auxiliary circuits, communication equipment, and relevant hardware should also be considered to pursue its future industrialization. Fourth, machine learning is a widely used AI technology in data processing, and the frontier of ML is deep learning, which can be used to classify and process a large amount of sensing data. The large amount and high randomness of sensing data captured by TENG sensor network put forward higher requirements for the accuracy of ML. New ML algorithm design, optimization, and training method need to be further developed to extract more feature information and capture the imperceptible sensing information. Finally, researchers need to devote to expand the application of AI such as digital twin. By combining TENG sensors with digital twins, the application scope of TENG will be highly expanded in smart manufacturing and engineering construction.
4. The application of TENG in IoTs keeps expanding in various scenarios. Previous studies have demonstrated the feasibility of TENG in smart agriculture, smart industry, smart city, emergency monitoring, and other fields. As an important part in the new-generation information technology, the IoTs have been widely used and are still under developing. Continuous research can explore more potential applications of TENG in logistics warehousing, positioning and navigation, intelligent buildings, public safety, enemy detection, etc.

Overall, as an energy harvester, TENG can collect a wide range of sustainable energy in the environment, which is very efficient and environmentally friendly. Besides, TENG itself can also be used as a self-powered sensor for various applications. Moreover, the applications of TENG sensors in smart IoTs have always been the main subject. The studies on the combination of AI technology based on TENG and IoTs are still in the early stage and need to be given with further attention. Although challenges still exist, the continuous research and exploration of multi-discipline intelligent applications based on TENGs with the assistance of AI technology will definitely shed light on the harmonious coexistence of humans and machines in the era of IoTs, as well as the immersive and efficient interaction in many scenes.
